# Using generalized linear models to implement g-estimation for survival data with time-varying confounding

**DOI:** 10.1002/sim.8997

**Published:** 2021-05-04

**Authors:** Shaun R. Seaman, Ruth H. Keogh, Oliver Dukes, Stijn Vansteelandt

**Affiliations:** 1 MRC Biostatistics Unit, University of Cambridge, Cambridge, UK; 2 Department of Medical Statistics, London School of Hygiene and Tropical Medicine, London, UK; 3 Department of Applied Mathematics, Computer Science and Statistics, Ghent University, Ghent, Belgium

**Keywords:** Aalen’s additive model, accelerated failure time model, causal effect, marginal structural model, structural nested cumulative failure time model, time-varying confounding

## Abstract

Using data from observational studies to estimate the causal effect of a time-varying exposure, repeatedly measured over time, on an outcome of interest requires careful adjustment for confounding. Standard regression adjustment for observed time-varying confounders is unsuitable, as it can eliminate part of the causal effect and induce bias. Inverse probability weighting, g-computation, and g-estimation have been proposed as being more suitable methods. G-estimation has some advantages over the other two methods, but until recently there has been a lack of flexible g-estimation methods for a survival time outcome. The recently proposed Structural Nested Cumulative Survival Time Model (SNCSTM) is such a method. Efficient estimation of the parameters of this model required bespoke software. In this article we show how the SNCSTM can be fitted efficiently via g-estimation using standard software for fitting generalised linear models.The ability to implement g-estimation for a survival outcome using standard statistical software greatly increases the potential uptake of this method. We illustrate the use of this method of fitting the SNCSTM by reanalyzing data from the UK Cystic Fibrosis Registry, and provide example R code to facilitate the use of this approach by other researchers.

## 1 Introduction

Observational studies in which exposures and confounders are repeatedly measured over time offer valuable opportunities for causal inference. Their temporal structure helps distinguish causes from effects, which makes adjustment for confounding more achievable than in comparable cross-sectional studies, but also more complicated. In particular, standard regression adjustment is generally unsuitable when we want to look at the joint effect of the repeatedly measured exposure on the outcome. This is because confounders of the association between exposure at one time and a later outcome of interest may lie on a causal pathway from an earlier exposure to the outcome. Standard regression adjustment eliminates that part of the latter exposure’s effect that operates via this pathway, as well as possibly introducing collider-stratification bias that can render exposure and outcome dependent even in the absence of a causal effect of exposure.^1^

This is an open access article under the terms of the Creative Commons Attribution License, which permits use, distribution and reproduction in any medium, provided the original work is properly cited.

Robins and Hernan (eg, Reference 2) introduced g-computation, g-estimation, and inverse probability weighting (IPW) methods to enable valid confounding adjustment in these complex longitudinal settings. With g-computation methods being very model-dependent and time-consuming, and g-estimation being relatively complicated, IPW methods have become the most popular of the three.^3^ However, attempting to answer the question that IPW methods address—namely, what would be the expected outcome if all individuals followed the same specific exposure trajectory?—may be overly ambitious in settings where that trajectory is implausible for some individuals. In these settings, the inverse probability weights are highly variable and the resulting estimate of expected outcome under certain exposure trajectories prone to large bias and variance. This issue is commonly addressed by truncating weights, which reduces variance but at the cost of increased bias. To overcome this concern, increasing attention has been devoted to estimation of the effect of less ambitious dynamic regimes (which consider (not) treating individuals only when (no) treatment is sufficiently likely based on their covariate data) or of the effect of shifting the observed exposure in some pre-defined manner.^4^ However, prespecification of interventions on which the observed data carry sufficient information can be a formidable task. The need for inverse weighting by the (joint) density of the exposure moreover continues to render results potentially sensitive to the tails of the exposure density, in particular complicating the analysis of continuous exposures. G-estimation methods are less ambitious, in that they estimate the effect of exposure at each time in strata of individuals with a specific exposure (and confounder) history at that time. This enables g-estimation methods to model effect modification by time-dependent covariates, but also to borrow information across strata. This borrowing of information explains why g-estimation methods tend to downweight strata of individuals who carry little information about the considered exposure effect. This, and the fact that g-estimation methods only require modelling of the exposure mean (as opposed to the density), tends to makes the resulting estimates more stable, especially when continuous exposures are of interest (in particular, weight truncation is unnecessary).

The uptake of g-estimation as a method for analyzing longitudinal observational data with continuous or count outcomes has recently been greatly facilitated by several articles that have shown how it can be implemented using standard regression software.^5–8^ Here we focus instead on survival time outcomes.

Structural Nested Accelerated Failure Time Models were introduced by Robins and Tsiatis.^9^ Allison et al^10^ and Sterne and Tilling^11^ provide R and STATA commands, respectively, for fitting these models. The model-fitting procedure involves an artificial recensoring step, in which originally uncensored failure times become censored. This step causes a loss of information and can lead to difficulties calculating the effect estimates, especially when the model involves more than one or two exposure effect parameters.^12^ For this reason, the models used in practice are usually very simple and do not explore interactions between exposures and covariates. The commands of Allison et al and Sterne and Tilling allow only for models with a single exposure effect parameter.

More recently, the more flexible Structural Nested Cumulative Failure Time Model^13^ and closely related Structural Nested Cumulative Survival Time Model (SNCSTM)^14,15^ have been developed. Dukes et al^14^ and Seaman et al^15^ (henceforth “SDKV”) discussed the relation between these two models and the relative advantages of the SNCSTM. These advantages include the existence of a closed-form parameter estimator and more automatic handling of random censoring. The Structural Nested Cumulative Failure Time Model parameterizes the causal effect of exposure on the probability of failure, but, as Picciotto et al^13^ noted, it is easily transformed into a model for the causal effect on the probability of survival. Dukes et al^14^ argued theoretically, and SDKV demonstrated, that when this transformation is made, reasonably efficient estimates of the causal effect are more easily attainable in SNCSTMs than in the model proposed by Picciotto et al. In this article, we focus on the SNCSTM. SDKV proposed three methods for fitting the SNCSTM. The first (which SDKV called “Method 1”) can be implemented using standard software for fitting generalized linear models (GLMs), but was shown to be considerably less efficient than the other two methods (“Method 2” and “Method 3”). Methods 2 and 3 were found to be roughly equally efficient, but Method 2 is easier to implement than Method 3, especially when exposure measurement times are irregular. In the situation where the exposure is only measured at baseline (a point exposure) Methods 2 and 3 are closely related to the semi-parametric efficient estimator of the causal effect of exposure.^14,15^ However, both methods have the drawback that they require bespoke software.

In this article, we show how the SNCSTM can be fitted efficiently using standard GLM software. Although Method 1 can also be applied using GLM software, the approach we propose in the current article is much more efficient; indeed, we prove that the resulting parameter estimates closely approximate those from Method 2 (Appendix F of Data S1). The accuracy of this approximation is demonstrated by reanalysing the data from the UK Cystic Fibrosis (CF) Registry that SDKV analysed. In Appendix G of Data S1 we provide example R code, to facilitate the use by other researchers of this method for efficiently fitting the SNCSTM using standard software.

The structure of this article is as follows. In Section 2 we consider the situation of a point exposure measured at baseline. Dukes et al^8^ described how standard software for fitting GLMs can be used to fit a multiplicative structural mean model for the probability of surviving to a single fixed post-baseline time. We adapt this method to fit models at multiple times simultaneously in an efficient way that accounts for the correlation between the survival indicators of the same individual at different times. In Section 3 we extend this to the setting in which an exposure is measured both at baseline and at one follow-up time, describe a simple SNCSTM for such data, and show how to estimate the causal effect parameters in this SNCSTM using standard GLM software. In both Sections 2 and 3 we assume, for simplicity, that all survival times are observed up to an administrative censoring time. Section 4 describes how to handle censoring times that differ between individuals. Section 5 describes more general SNCSTMs that allow for exposures measured at more than two times, and for modification by previously measured variables of the causal effects of exposure. In Section 6 we provide an estimator of the survivor function when all exposures are set to zero. Section 7 shows the results of our re-analysis of the CF data.

## 2 Estimating The Effect of a Point Exposure

Consider a study in which an exposure *A* and a set of variables *L* are measured at time *t* = 0 on each of a random sample of *n* individuals, and let *T* denote an individual’s failure time. Exposure could be binary (eg, high/low dose of radiation) or continuous (eg, actual dose of radiation). We shall use the subscript *i* where necessary to index the individual in the sample (*i* = 1, …, *n*).

We denote by *T*(0) the failure time that an individual would have if his exposure were set to zero by an intervention. This is often called the “potential” or “counterfactual” failure time. We make the “no unmeasured confounders” assumption that *L* is sufficient to adjust for confounding, in the sense that *T*(0) is conditionally independent of *A* given *L*. We also assume that *T* = *T*(0) for individuals with observed values of *A* equal to zero. This so-called consistency assumption is justified when the intervention of setting exposure to zero has no effect in individuals whose exposure is naturally zero.

We assume that

(1) $$\frac{P\{T(0)\geq t|A,L\}}{P(T\geq t|A,L)}=\exp(A\psi t)\;(t>0),$$

where *ψ* is an unknown parameter. Equation (1) states that the conditional probability of surviving to time *t* given *A* and *L* is multiplied by exp(*Aψt*) when *A* is set to zero. In particular, exp(*ψt*) expresses the effect, on the relative risk scale, of removing exposure (ie, setting it equal to zero) on the chances of surviving to time *t* of an individual whose observed exposure equals one. A positive value of *ψ* implies that exposure is harmful (because reducing exposure increases the probability of survival); a negative value, that it is beneficial. Note that we are assuming, for simplicity, that the causal effect of exposure on the survival time is the same on the relative risk scale (ie, *ψ*) regardless of the value of *L*. In Section 5 we shall relax this assumption and allow the causal effect to depend on *L*.

Note that if the consistency assumption and the model of Equation (1) were strengthened in the way that we shall describe in the next two sentences, then *ψ* could be given a more general interpretation. First, the consistency assumption that *T* = *T*(0) for individuals with *A* = 0 would be replaced by the stronger assumption that *T* = *T*(*A*) for all individuals, where *T*(*a*) is the (potential) failure time that an individual would have if his exposure were set to *a* by an intervention. Second, the no unmeasured confounders assumption would be strengthened to *T*(*a*) being conditionally independent of *A* given *L* for all (feasible) values *a* of *A*, so that Equation (1) would be replaced by *P*{*T*(0) ≥ *t|L*}/*P*{*T*(*a*) ≥ *t*|*L*} = exp(*aψt*). If these stronger assumptions were made, then exp(*ψt*) would describe the effect on survival of any individual of intervening to reduce his exposure by one unit.

By taking logs of each side of Equation (1) and differentiating with respect to *t*, it can be shown that Model (1) can be written equivalently as

(2) $$h_{T}(t|A,L)=h_{T(0)}(t|A,L)+A\psi \;(t>0),$$

where *h_T_*(*t* | *A*, *L*) is the conditional hazard of *T* given *A* and *L* at time *t*, and *h_T_*_(0)_(*t* | *A*, *L*) is the conditional hazard of *T*(0). Thus, *ψ* also describes the change in hazard per unit of exposure when the exposure is set to zero by an intervention.

The exposure has an additive effect on the hazard, and *ψ* describes a hazard difference. In this model, the function *h_T_*_(0)_(*t* | *A*, *L*) is left unspecified.

The consistency assumption and the assumption that *T*(0) is independent of *A* given *L* mean that *h_T_*_(0)_(*t* | *A*, *L*) = *h_T_*(*t* | *A* = 0, *L*), and hence Equation (2) can also be written as *h_T_*(*t*| *A* = 0*, L*) = *h_T_*(*t*| *A* = 0, *L*) + *ψA*. This is closely related to the Aalen additive hazards model with constant exposure effect,^16,17^ but is more general in that the Aalen model makes the additional assumption that, for any *t >* 0, *h_T_*(*t* | *A* = 0, *L*) is a simple additive function of the variables *L*.

For each time *t*, Model (1) defines a so-called multiplicative structural mean model^18^ for the probability of surviving to that time. It differs from more common multiplicative models for risk in that it only parameterizes the exposure effect of interest, and not the effect of confounders. This turns out to be important when addressing time-varying confounding, in order to avoid assuming incompatible models.^15^ Dukes et al^8^ showed how multiplicative structural mean models, such as Model (1), can be fitted at a single time *t* by using standard software for fitting GLMs. To fit Model (1) specifically, the procedure is as follows.

We refer to *E*(*A* | *L*) as the “propensity score” (this generalizes the usual definition of a propensity score to include continuous exposures).^19^ We specify a model for this propensity score, for example, a linear regression model if *A* is continuous, or a logistic regression model if *A* is binary. Fit this propensity score model to the sample, and denote as *ê*(*L*) the resulting fitted value of *A* for an individual with covariate value *L*. Then, for the given time *t*, fit the GLM with gamma distribution, log link function, covariate −{*A* − *ê*(*L*)}*t*, no intercept, and outcome variable *I*(*T* ≥ *t*) to the sample, that is, the model that assumes log *E* (*I*(*T* ≥ *t*)) = −*ψ*{*A* − *ê*(*L*)}*t*. The resulting estimate $\tilde{\psi }$ of the coefficient *ψ* in this gamma GLM is a consistent estimator of the parameter *ψ* in Model (1) for the given time *t*, for the reason given in the next paragraph.

The no unmeasured confounders assumption implies that *P*{*T*(0) ≥ *t* | *A*, *L*} does not depend on *A*. It then follows from Model (1) that *P*(*T* ≥ *t*|*A, L*) exp(*Aψt*) also does not depend on *A*. A way to estimate *ψ* is therefore to find the value of *ψ* that makes the ‘blipped’ survival indicator *I*(*T* ≥ *t*) exp(*Aψt*) conditionally uncorrelated with *A* given *L*. This method is known as “g-estimation.”^20^ The estimate $\tilde{\psi }$ achieves this zero correlation (in large samples). This is because the estimating equation of the gamma GLM described in the last paragraph is

(3) $$\overset{n}{\underset{i=1}{\sum^{\text{​}}}}\left\{A_{i}-\hat{e}\left(L_{i}\right)\right\}\times \left(I\left(T_{i}\geq t\right)\exp\left[\tilde{\psi }\left\{A_{i}-\hat{e}\left(L_{i}\right)\right\}t\right]-1\right)=0$$

and so $\tilde{\psi }$ converges to the value of *ψ* that solves E[{A−E(A∣L)}×(I(T≥t)exp[ψ{A−E(A∣L)}t]−1)∣L]=0 or equivalently solves E({A−E(A∣L)}×I(T≥t)exp(ψAt)∣L)=0 A more formal proof is given in Appendix I of Data S1.

Although $\tilde{\psi }$ is a consistent estimator of *ψ*, it depends on the choice of time *t*, which is arbitrary. It is also inefficient, because $\tilde{\psi }$ depends on the survival time *T* only through the survival indicator *I*(*T* ≥ *t*) at a single value of *t*. It is more efficient to fit Model (1) at multiple times *t* simultaneously. Although generalized estimating equations can be used to do this, this strategy does not make efficient use of the data, because the indicators of surviving to the multiple times are highly correlated. So instead, in the next paragraph we propose a more efficient method, which uses survival indicators that are independent of one another.

It follows from Model (1) that, for any *δ >* 0,

(4) $$\frac{P\{T(0)\geq t+\delta |A,L,T(0)\geq t\}}{P\{T\geq t+\delta |A,L,T\geq t\}}=\exp(A\psi \delta ).$$

For a given *δ* and *t*, Model (4) also defines a multiplicative structural mean model, but now for the probability of surviving to time *t* + *δ* among individuals who have survived to time *t*. Our approach is to fit Model (4) at multiple times *t* simultaneously, using standard software for fitting a GLM with gamma distribution. The steps of the procedure are as follows. We assume for now that all failure times are observed, except those greater than some common administrative censoring time *C*, and that we have chosen to fit Model (4) over, say, 20 equally spaced time points *t*. The first three steps involve creating an expanded dataset in which each individual can appear multiple times.

1. Set *δ* = *C*/20 and create 21 copies of each of the *n* sampled individuals.
2. Introduce a time variable *Q* and set *Q* = 0 for the first copy of each individual, *Q* = *δ* for the second copy, *Q* = 2*δ* for the third, *Q* = 3*δ* for the fourth, and so on; the 21st copy has *Q* = *C*.
3. Discard all copies for which the failure time *T* is less than the value of *Q*, and call the remaining copies ‘pseudo-individuals’.
  For example, if *C* = 10, then *δ* = 0.5 and an individual with *T* = 3.7 yields eight pseudo-individuals, with *Q* = 0, 0.5, 1, …, 3.5. This reflects the fact that this individual contributes information about Model (4) at eight time points, *t* = 0, 0.5, 1, …, 3.5.
4. Specify a canonical GLM for the propensity score *E*(*A* | *L*), for example, a linear or logistic regression model. Fit this GLM for *A* given *L*, but including *Q* as a single extra covariate, to the full set of pseudo-individuals. Let *ê*(*L, Q*) be the resulting fitted value of *A*, and let $\overset{\wedge }{\Delta }=\overset{\wedge }{\Delta }(L,Q)=A-\hat{e}(L,Q)$ be the residual.
  The fitted value *ê*(*L, t*) is an estimate of *e*(*L*, *t*) = *E*(*A* | *L*, *T* ≥ *t*), the expected exposure given *L* and survival to time *t*. This way of estimating *e*(*L*, *t*) is justified by the fact (proved by SDKV) that Model (2) implies that the distribution of *A* given *L* and *T* ≥ *t* obeys the same canonical GLM as that specified for *A* given *L*, but with the intercept shifted by *t* times a constant.
5. Fit the GLM with gamma distribution and log link function to the set of pseudo-individuals who have *Q* + *δ* ≤ *C*. This GLM uses the single covariate $-\overset{\wedge }{\Delta }\delta$ and has no intercept, and the outcome variable is the indicator, *I*(*T* ≥ *Q* + *δ*), of surviving to time *Q* + *δ*. Let $\tilde{\psi }$ denote the resulting estimate of the coefficient of $-\overset{\wedge }{\Delta }\delta$ in this model.

Provided that the GLM for the propensity score *E*(*A* | *L*) is correctly specified, $\tilde{\psi }$ is a consistent estimator of *ψ*. This follows from the same argument as that given above for the consistency of $\tilde{\psi }$ when Model (1) is fitted at a single time *t* (see Appendix E of Data S1 for a formal proof).

In the above description, we set *δ* = *C*/20, and so fitted Model (4) over 20 time points. This choice is somewhat arbitrary; more generally, we could choose *δ* = *C*/*m* for any positive integer *m*, in which case there are *m* time points. The estimate $\tilde{\psi }$ depends on *δ*, but provided *δ* is sufficiently small that the proportions of all observed failures that occur during each of the time intervals [0*,δ*], [*δ*, 2*δ*], …, [*C* − *δ, C*] are small (eg, less than 10% of the failures), any further reduction in *δ* will make little difference to $\tilde{\psi }$. Also, as we prove in Appendix F of Data S1 and demonstrate in Section 7, when *δ* is small $\tilde{\psi }$ closely approximates the estimate obtained by SDKV’s Method 2.

The SE of $\tilde{\psi }$ can be estimated using a robust sandwich estimator of the coefficient of $-\overset{\wedge }{\Delta }\delta$ in the gamma GLM. However, this ignores the uncertainty in the estimate $\overset{\wedge }{\Delta }$ of *A* − *E*(*A* | *L*, *T* ≥ *t*) and tends to overestimate the SE of $\tilde{\psi }$.^8^ For this reason, and because this approach does not work when, as in Section 3, exposure is measured at multiple times, we use bootstrap to estimate SEs.

## 3 Estimating The Joint Effect of Two Exposures

Now suppose the exposure and confounders are measured at time *t* = 0 and again at time *s*_1_ in those who have not failed before time *s*_1_. Denote the exposure and confounders at time 0 as *A*_0_ and *L*_0_, and those at time *s*_1_ as *A*_1_ and *L*_1_. Continue to assume that the only censoring is administrative and takes place at time *C* (with *C > s*_1_). Let Ā_1_ = (*A*_0_ *, A*_1_) and ${\bar{L}}_{1}=\left(L_{0},L_{1}\right)$.

**Figure 1:**
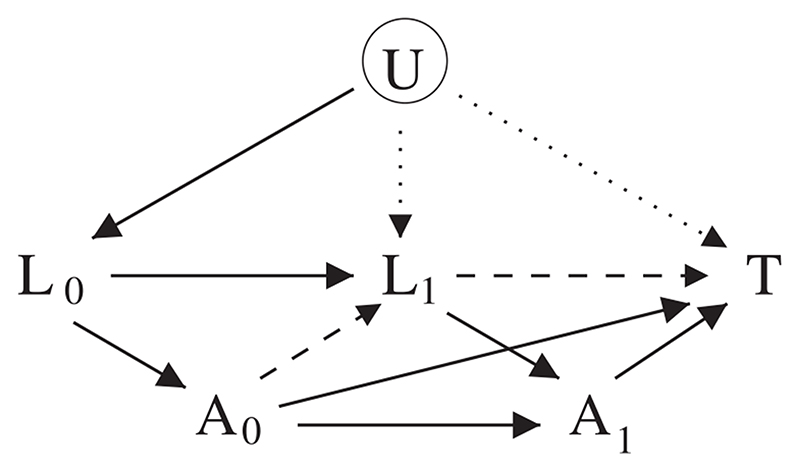
Causal graph showing exposure (*A*_0_ and *A*_1_) and confounders (*L*_0_ and *L*_1_) measured at baseline and at follow-up, a survival time *T*, and a latent variable *U*. Regression adjustment for *L*_1_ eliminates the indirect causal effect of *A*_0_ on *T* mediated by *L*_1_ (shown by the broken arrows) and causes collider stratification bias by unblocking the path from *A*_0_ to *T* via *U* (shown by the dotted arrows)

Estimating the joint effect of *A*_0_ and *A*_1_ on *T* is not straightforward. The problem with standard regression adjustment is that if we do not adjust for *L*_1_, the association between *A*_1_ and *T* is confounded, but if we do adjust for *L*_1_, the indirect effect of *A*_0_ that operates via its effect on *L*_1_ will be adjusted away. In addition, if there are common causes of *L*_1_ and *T*, ‘collider stratification’ bias may be induced. This problem is shown in Figure 1. The SNCSTM of SDKV is one way to estimate the joint causal effect of *A*_0_ and *A*_1_. In this section we describe the SNCSTM and explain how it can be fitted using standard GLM software.

### 3.1 The causal effect of *A*_1_

Estimating the causal effect of *A*_1_ poses no particular challenges: *A*_1_ can be viewed as a point exposure, measured at time *s*_1_, and thus the methods from the previous section are readily applicable. In particular, let *T*(*A*_0_, 0) be the failure time when *A*_1_ is set to zero by an intervention at time *s*_1_. Note that if the individual does not survive to time *s*_1_, *T*(*A*_0_, 0) equals *T*. We make the consistency assumption that *T* = *T*(*A*_0_, 0) for individuals with observed values of *A*_1_ equal to zero. We also make the no unmeasured confounders assumption that *A*_0_ and ${\bar{L}}_{1}$ are sufficient to adjust for confounding of the causal effect of *A*_1_, in the sense that *T*(*A*_0_, 0) is independent of *A*_1_ given *A*_0_, ${\bar{L}}_{1}$ and *T* ≥ *s*_1_. The causal effect of *A*_1_ on the hazard of failure can be parameterized as

(5) $$h_{T\left(A_{0},0\right)}\left(t|{\bar{A}}_{1},{\bar{L}}_{1}\right)=h_{T}\left(t|{\bar{A}}_{1},{\bar{L}}_{1}\right)-A_{1}\psi_{1}\;\text{if}\;t\geq s_{1},$$

where ψ_1_ is an unknown parameter and $h_{T\left(A_{0},0\right)}\left(t|A_{1},{\bar{L}}_{1}\right)$ is the (conditional) hazard corresponding to the failure time *T*(*A*_0_, 0) (given ${\bar{A}}_{1},{\bar{L}}_{1}$). As in Section 2, we are assuming here that the causal effect of *A*_1_ does not depend on the history (*A*_0_, ${\bar{L}}_{1}$). This assumption will be relaxed in Section 5, where the general SNCSTM is described. Model (5) implies

(6) $$\frac{P\left\{T\left(A_{0},0\right)\geq t|{\bar{A}}_{1},{\bar{L}}_{1},T\geq s_{1}\right\}}{P\left\{T\geq t|{\bar{A}}_{1},{\bar{L}}_{1},T\geq s_{1}\right\}}=\exp\left\{A_{1}\psi_{1}\left(t-s_{1}\right)\right\}\;\text{if}\;t\geq s_{1}.$$

Estimation of *ψ*_1_ is readily done using the method described for *ψ* in the previous section upon letting *A*_1_ and (*A*_0_, ${\bar{L}}_{1}$) play the roles of *A* and *L*, respectively. First, create the pseudo-individuals as described in Section 2. Assume, for simplicity, that the value of *δ* has been chosen so that*s*_1_ and *C* are multiples of *δ*, so that pseudo-individuals with *Q* = 0*, δ*, 2*δ*, … *, s*_1_ *, s*_1_ + *δ*, … *, C* are created. Specify a canonical GLM for *A*_1_ given *A*_0_ and ${\bar{L}}_{1}$ and *T* ≥ *s*_1_. Fit this GLM with (*Q* − *s*_1_) included as an extra covariate to the set of pseudo-individuals with *Q* ≥ *s*_1_. The resulting fitted values *ê*_1_ of *A*_1_ are estimates of $e_{1}\left(A_{0},{\bar{L}}_{1},Q\right)=E\left(A_{1}|A_{0},{\bar{L}}_{1},T\geq Q\right)$. Calculate ${\overset{\wedge }{\Delta }}_{1}=A_{1}-{\hat{e}}_{1}$ for each pseudo-individual with *Q* ≥ *s*_1_. Finally, fit the gamma GLM with covariate $-{\overset{\wedge }{\Delta }}_{1}\delta$ and no intercept to the set consisting of those pseudo-individuals with *Q* ≥ *s*_1_ and *Q* + *δ* ≤ *C*. Let ${\hat{\psi }}_{1}$ denote the resulting estimator.

### 3.2 The causal effect of *A*_0_

Estimating the causal effect of *A*_0_ is more subtle for the following reason. If it were the case, for example, that a change in *A*_0_ affects the failure time only by changing *A*_1_, it would be desirable to know that *A*_0_ has *no additional causal effect* on failure time. The causal effect of *A*_0_ will therefore be defined as a controlled direct effect, setting *A*_1_ to zero. In particular, let *T*(0) be the failure time when *A*_0_ and *A*_1_ are both set to zero at times 0 and *s*_1_ respectively. Then the (controlled direct) causal effect of *A*_0_ on the hazard of failure can be parameterised as

(7) $$h_{T(0)}\left(t|A_{0},L_{0}\right)=\{\begin{array}{ll} h_{T\left(A_{0},0\right)}\left(t|A_{0},L_{0}\right)-A_{0}\psi_{0(0)} & \text{if}\;t<s_{1}\\ h_{T\left(A_{0},0\right)}\left(t|A_{0},L_{0}\right)-A_{0}\psi_{0(1)} & \text{ if }t\geq s_{1} \end{array}$$

where *h_T_*_(0)_(*t* | *A*_0_, *L*_0_) is the hazard corresponding to *T*(0) (given *A*_0_, *L*_0_), and *ψ*_0(0)_ and *ψ*_0(1)_ are unknown parameters. The first line of Equation (7) means that setting *A*_0_ to zero reduces the hazard (given *A*_0_ and *L*_0_) by *ψ*_0(0)_ *A*_0_ prior to time *s*_1_. The second line means that setting *A*_0_ to zero, when *A*_1_ is already set to zero, reduces the hazard after time *s*_1_ by *ψ*_0(1)_ *A*_0_. Thus, *ψ*_0(0)_ and *ψ*_0(1)_ describe causal effects of *A*_0_ before and after time *s*_1_, respectively; the first is the “immediate” effect, the second a “delayed” effect. Here, conditioning on *L*_0_ is motivated by the no unmeasured confounders assumption, which we henceforth make, that *L*_0_ is sufficient to adjust for confounding of the causal effect of *A*_0_, in the sense that *T*(0) is independent of *A*_0_ given *L*_0_. Model (7) implies

(8) $$\frac{P\left\{T(0)\geq t|A_{0},L_{0}\right\}}{P\left\{T\left(A_{0},0\right)\geq t|A_{0},L_{0}\right\}}=\{\begin{array}{ll} \exp\left(A_{0}\psi_{0(0)}t\right) & \text{if}\;t\leq s_{1}\\ \exp\left\{A_{0}\psi_{0(0)}s_{1}+A_{0}\psi_{0(1)}\left(t-s_{1}\right)\right\} & \text{if}\;t>s_{1} \end{array}$$

We make the consistency assumption that *T* = *T*(0) for those individuals whose observed values of *A*_0_ and *A*_1_ equal zero. Estimation of *ψ*_0(0)_ is readily done using the method described for *ψ* in Section 2 upon letting *T*(*A*_0_, 0), *A*_0_, and *L*_0_ play the roles of *T*, *A*, and *L*, respectively, and specifying a canonical GLM for *A*_0_ given *L*_0_. We can do this because exposure *A*_1_ is irrelevant until time *s*_1_ (making the events *T*(*A*_0_, 0) ≥ *t* and *T* ≥ *t* equivalent for *t* ≤ *s*_1_). The procedure is as follows. Fit the GLM for *A*_0_ given *L*_0_ with *Q* included as an extra covariate to the set of pseudo-individuals with *Q* ≤ *s*_1_. This provides an estimate *ê*_0_ of *e*_0_(*L*_0_, *Q*), where *e*_0_(*L*_0_, *t*) = *E*(*A*_0_ | *L*_0_, *T* ≥ *t*) for *t* ≤ *s*_1_. Then fit the gamma GLM with log link function, covariate $-{\overset{\wedge }{\Delta }}_{0}\delta$, no intercept and outcome *I*(*T* ≥ *Q*) to the pseudo-individuals with *Q* + *δ* ≤ *s*_1_, where ${\overset{\wedge }{\Delta }}_{0}=A_{0}-{\hat{e}}_{0}$. Let ${\hat{\psi }}_{0(0)}$ be the resulting estimator.

Estimating *ψ*_0(1)_ is slightly more complicated, because fitting Model (7) for *t > s*_1_ requires data on *T*(*A*_0_, 0) (since the events *T*(*A*_0_, 0) ≥ *t* and *T* ≥ *t* are not equivalent for *t > s*_1_). If *T*(*A*_0_, 0) were observed, the procedure of the previous paragraph could be used, upon replacing *T* by *T*(*A*_0_, 0). This would involve fitting a gamma GLM with indicator *I*{*T*(*A*_0_, 0) ≥ *Q* + *δ*} as outcome variable to the pseudo-individuals with *T*(*A*_0_, 0) ≥ *Q*. Because *T*(*A*_0_, 0) is unobserved, we shall instead fit the gamma GLM with outcome variable $I(T\geq Q+\delta )\exp\left({\hat{\psi }}_{1}A_{1}\delta \right)$ to the pseudo-individuals with *T* ≥ *Q*, with each pseudo-individual being weighted by a factor $\exp\left\{A_{1}{\hat{\psi }}_{1}\left(Q-s_{1}\right)\right\}$. Here, the term $\exp\left({\hat{\psi }}_{1}A_{1}\delta \right)$ blips down the effect of the observed exposure *A*_1_ over the time window from *Q* to *Q* + *δ*, as justified by Model (6), and the weight $\exp\left\{A_{1}{\hat{\psi }}_{1}\left(Q-s_{1}\right)\right\}$ reflects the fact that the frequencies of the events *T*(*A*_0_, 0) ≥ *Q* and *T* ≥ *Q* differ by this factor. This same weighting is also required when estimating *e*_0_(*L*_0_, *Q*), where *e*_0_(*L*_0_, *t*) = *E*{*A*_0_ | *L*_0_, *T*(*A*_0_, 0) ≥ *t*} for *t* ≥ *s*_1_. In more detail, the procedure is as follows.

First, fit the GLM for *A*_0_ given *L*_0_ with extra covariate *Q* to the pseudo-individuals with *Q* ≥ *s*_1_ using weights $\exp\left\{A_{1}{\hat{\psi }}_{1}\left(Q-s_{1}\right)\right\}$. The resulting fitted values *ê*_0_ of *A*_0_ are estimates of *e*_0_(*L*_0_, *Q*). Let ${\overset{\wedge }{\Delta }}_{0}=A-{\hat{e}}_{0}$. Then fit the gamma GLM with log link, single covariate $-{\overset{\wedge }{\Delta }}_{0}\delta$, no intercept, and outcome variable $I(T\geq Q+\delta )\exp\left({\hat{\psi }}_{1}A_{1}\delta \right)$ to the pseudo-individuals with *Q* ≥ *s*_1_ and *Q* + *δ* ≤ *C* and using weights $\exp\left\{A_{1}{\hat{\psi }}_{1}\left(Q-s_{1}\right)\right\}$. let ${\hat{\psi }}_{0(1)}$ be the resulting estimate of the coefficient of$-{\overset{\wedge }{\Delta }}_{0}\delta$ Appendix E of Data S1 contains a proof that ${\hat{\psi }}_{0(1)}$ is a consistent estimator of *ψ*_0(1)_.

The Weights $\exp\left\{A_{1}{\hat{\psi }}_{1}\left(Q-s_{1}\right)\right\}$, used above, are different from the inverse probability of exposure weights used to fit MSMs, and do not suffer from the instability that can plague the latter weights. In many applications, the causal effect, *ψ*_1_, of *A*_1_ is small, so that $\exp\left\{A_{1}{\hat{\psi }}_{1}\left(Q-s_{1}\right)\right\}$ should be quite close to 1 for all pseudo-individuals.

We recommend choosing *δ* to be small enough that no more than 10% of the failures observed to occur before time *s*_1_ happen during any one of the time intervals [0*, δ*], [*δ*, 2*δ*], …, [*s*_1_ − *δ, s*_1_], and no more than 10% of those observed to occur after time *s*_1_ happen during one of intervals [*s*_1_ *, s*_1_ + *δ*], … , [*C* − *δ, C*].

In some applications, it may be reasonable to assume *A*_0_ and *A*_1_ have the same immediate effect on survival, in the sense that *ψ*_0(0)_ = *ψ*_1_. This common parameter can be estimated by stacking the two expanded datasets to which the gamma GLMs for *ψ*_0(0)_ and *ψ*_1_ would be fitted and instead fitting a single gamma GLM to the stacked set. The covariate in this single GLM equals $-{\overset{\wedge }{\Delta }}_{0}\delta$ for pseudo-individuals with *Q < s*_1_ and equals $-{\overset{\wedge }{\Delta }}_{1}\delta$ for those with *Q* ≥ *s*_1_.

## 4 Censoring

Hitherto we have assumed the censoring time *C* is fixed and the same for everyone. In practice, censoring times may vary, because individuals may enter the study at different dates and be followed up to the same date and/or some individuals may drop out before the end of the study. With two modifications, the estimation method described above remains valid, provided that the hazard of censoring at each time *t* (among uncensored survivors at that time) has no residual dependence on the actual failure time *T* or the histories of the exposure and confounders up to time *T*, given the baseline confounders *L*_0_. The pseudo-individuals with *Q* = 0*, δ*, 2*δ*, … are still created from each individual, where *δ* is the same for all individuals. The first modification is that *T* must be redefined as the minimum of the failure time and censoring time. This means, in particular, that pseudo-individuals with *Q > C* are discarded. The second is that when fitting the gamma GLMs, any pseudo-individual whose survival status at time *Q* + *δ* is unknown, that is, any pseudo-individual with *C* < *T* and *Q* + *δ > C*, must be omitted.

If the aforementioned hazard of censoring at time *t* further depends on the history of exposures and confounders up to time *t* but has no residual dependence on future exposures and confounders or the actual failure time, inverse probability of censoring weights can be used. See Appendix C of Data S1for details.

## 5 The General Sncstm

The causal effects of *A*_0_ and *A*_1_ can be allowed to depend on functions of *L*_0_ and (*A*_0_, ${\bar{L}}_{1}$), respectively. For example, to allow them to be modified by, respectively, *L*_0_ and *L*_1_, we could replace Equations (5) and (7) by

$$h_{T\left(A_{0},0\right)}\left(t|{\bar{A}}_{1},{\bar{L}}_{1}\right)=h_{T}\left(t|{\bar{A}}_{1},{\bar{L}}_{1}\right)-A_{1}\psi_{1}^{0}-A_{1}L_{1}\psi_{1}^{L}\;\text{if}\;t\geq s_{1}$$

$$h_{T(0)}\left(t|A_{0},L_{0}\right)=\{\begin{array}{ll} h_{T\left(A_{0},0\right)}\left(t|A_{0},L_{0}\right)-A_{0}\psi_{0(0)}^{0}-A_{0}L_{0}\psi_{0(0)}^{L} & \text{if}\;t<s_{1}\\ h_{T\left(A_{0},0\right)}\left(t|A_{0},L_{0}\right)-A_{0}\psi_{0(1)}^{0}-A_{0}L_{0}\psi_{0(1)}^{L} & \text{if}\;t\geq s_{1} \end{array}$$

This is equivalent to replacing Equations (6) and (8) by

$$\frac{P\left\{T\left(A_{0},0\right)\geq t|{\bar{A}}_{1},{\bar{L}}_{1},T\geq s_{1}\right\}}{P\left\{T\geq t|A_{1},{\bar{L}}_{1},T\geq s_{1}\right\}}=\exp\left\{A_{1}\left(\psi_{1}^{0}+L_{1}\psi_{1}^{L}\right)\left(t-s_{1}\right)\right\}\;\text{if}\;t\geq s_{1}$$

$$\frac{P\left\{T\left(A_{0},0\right)\geq t|{\bar{A}}_{1},{\bar{L}}_{1},T\geq s_{1}\right\}}{P\left\{T\geq t|{\bar{A}}_{1},{\bar{L}}_{1},T\geq s_{1}\right\}}=\{\begin{array}{ll} \exp\left\{A_{0}\left(\psi_{0(0)}^{0}+L_{0}\psi_{0(0)}^{L}\right)t\right\} & \text{if}\;t\leq s_{1}\\ \exp\{A_{0}\left(\psi_{0(0)}^{0}+L_{0}\psi_{0(0)}^{L}\right)s_{1}+A_{0}\left(\psi_{0(1)}^{0}+L_{0}\psi_{0(1)}^{L}\right)\left(t-s_{1}\right)\} & \text{if}\;t>s_{1} \end{array}$$

Now $\psi_{0(0)}^{0}$ is the causal effect of a unit decrease in exposure *A*_1_ for an individual with unit exposure and *L*_1_ = 0, and $\psi_{1}^{0}$ describes how much this causal effect differs for an individual with nonzero *L*_1_. Similarly, $\psi_{1}^{L}$ and $\psi_{0(1)}^{0}$ are the causal effects of reducing *A*_0_ in individuals with *L*_0_ = 0, and $\psi_{0(0)}^{L}$ and $\psi_{0(1)}^{L}$ describe how those effects vary according to *L*_0_.

This model can be fitted using the method of Section 3 with three simple modifications. First, the assumed GLM for *A*_0_ given *L*_0_ is fitted with both *Q* and *L*_0_ *Q* as extra covariates. Similarly, the GLM for *A*_1_ given *A*_0_, ${\bar{L}}_{1}$ and *T* ≥ *s*_1_ is fitted with both (*Q* − *s*_1_) and *L*_1_(*Q* − *s*_1_) as extra covariates. Second, the single covariate $-{\overset{\wedge }{\Delta }}_{0}\delta$ in the gamma GLM previously used to estimate *ψ*_0(0)_ is replaced by covariates $-{\overset{\wedge }{\Delta }}_{0}\delta$ and $-L_{0}{\overset{\wedge }{\Delta }}_{0}\delta$. Their estimated coefficients are now consistent estimates of $\psi_{0(0)}^{0}$ and $\psi_{0(0)}^{L}$. Analogous modifications are made when fitting the two gamma GLMs previously used to estimate *ψ*_1_ and *ψ*_0(1)_ respectively. Third, when fitting the gamma GLM previously used to estimate *ψ*_0(1)_, the weights are now $\exp\{A_{1}({\hat{\psi }}_{1}^{0}+L_{1}{\hat{\psi }}_{1}^{L})\left(Q-s_{1}\right)\}$ and the outcome is $I(T\geq Q+\delta )\exp\left\{A_{1}\left({\hat{\psi }}_{1}^{0}+L_{1}{\hat{\psi }}_{1}^{L}\right)\delta \right\}$. The estimating equations solved when fitting these three modified gamma GLMs are given in Appendix J of Data S1.

The SNCSTM easily extends to more than two time points. Here we consider the case without effect modification; effect modification is handled just as in the last paragraph. Let *A_k_* and *L_k_* (*k* = 0, …, *K*) be the exposure and confounders measured at time *s_k_* (0 = *s*_0_ *< s*_1_ *<* … *< s_K_*), and let Ā_k_ = (*A*_0_, … *, A_k_*) and ${\bar{L}}_{k}=\left(L_{0},\ldots ,L_{k}\right)$. Let *T*(Ā_k_, 0) be the failure time when *A_k_*_+1_, …, *A_K_* are set to zero by intervention, and assume Ā_k−1_ and ${\bar{L}}_{k}$ are sufficient to adjust for confounding, in the sense that *T*(Ā_k−1_, 0) is independent of *A_k_* given Ā_k−1_, ${\bar{L}}_{k}$ and *T* ≥ *s_k_*. Also assume consistency: *T* = *T*(Ā_k−1_, 0) for all individuals whose observed values of *A_k_*, *A_k_*_+1_, …, *A_K_* equal zero. Let $h_{T\left({\bar{A}}_{k},0\right)}\left(t|{\bar{A}}_{k},{\bar{L}}_{k}\right)\left(\text{for}\;t\geq s_{k}\right)$ be the hazard at time *t* of *T*(Ā_k_, 0) given Ā_k_ and ${\bar{L}}_{k}$. The SNCSTM assumes that this hazard is related to the hazard when *A_k_* is also set to zero by

$$h_{T\left(A_{k-1},0\right)}\left(t|{\bar{A}}_{k},{\bar{L}}_{k}\right)=h_{T\left(A_{k},0\right)}\left(t|{\bar{A}}_{k},{\bar{L}}_{k}\right)-A_{k}\psi_{k(l)},$$

when *s_l_* ≤ *t < s_l_*_+1_. The parameter *ψ_k_*_(*l*)_ is the causal effect of *A_k_* on the hazard between times *s_l_* and *s_l_*_+1_. This model implies

(9) $$\frac{P\left\{T\left({\bar{A}}_{k-1},0\right)\geq t|{\bar{A}}_{k},{\bar{L}}_{k},T\geq s_{k}\right\}}{P\left\{T\left({\bar{A}}_{k},0\right)\geq t|{\bar{A}}_{k},{\bar{L}}_{k},T\geq s_{k}\right\}}=\exp\left\{\overset{l-1}{\underset{j=k}{\sum^{\text{​}}}}A_{k}\psi_{k(j)}\left(s_{j+1}-s_{j}\right)+A_{k}\psi_{k(l)}\left(t-s_{l}\right)\right\}$$

when *s_l_* ≤ *t < s_l_*_+1_. The model in Section 3 is a special case of this, with *K* = 1 and *ψ*_1(1)_ written as *ψ*_1_.

Estimation of *ψ_k_*_(*k*)_ (*k* = 0, …, *K*) proceeds in the same way as for *ψ*_0(0)_ and *ψ*_1_ in Section 3. Estimation of *ψ_k_*_(*k*+1)_ (*k* = 0, …, *K* − 1) is like that of *ψ*_0(1)_, and estimation of the remaining parameters *ψ_k_*_(*k*+2)_ etc. is a simple extension of this.

So far, we have assumed the exposure and confounder measurement times, *s*_0_, …, *s_K_*, are the same for all individuals. We now briefly describe the two modifications needed to estimate *ψ_k_*_(*l*)_ when these times vary. For simplicity, we assume no effect modification. First, the pseudo-individuals are created as follows. From each individual with *T* ≥ *s_l_* and for each value of *t* = *s_k_, s_k_* + *δ, s_k_* + 2*δ*, … that satisfies *s_l_* ≤ *t* ≤ *s_l_*_+1_ and *t* ≤ *T*, create a pseudo-individual with *Q* = *t*. Second, if *l > k*, include extra covariates (*s_k_*_+1_ − *s_k_*), (*s_k_*_+2_ − *s_k_*_+1_), …, (*s_l_* − *s_l_*_−1_) when fitting the GLM for *A_k_* given (Ā_k−1_, ${\bar{L}}_{k}$).

See Appendices A and B of Data S1 for more details of the general SNCSTM and how to fit it using gamma GLMs, including in the situation where *s*_0_, …, *s_K_* can vary between individuals or where the measurement times are common but are not multiples of *δ*. In Appendix H of Data S1 we describe how to fit several more SNCSTMs using gamma GLMs. These SNCSTMs include models for a categorical exposure with more than two levels, models in which the causal effect of exposure varies during the intervals between exposure measurement times, and models in which the causal effect of a continuous exposure is nonlinear.

## 6 Estimating Survival Probability When All Exposures Are Set to Zero

Interpretation of the results from fitting the SNCSTM is often helped by visualizing the probability of survival to time *t* when *A*_0_, …, *A_K_* are all set to zero, that is, *P*{*T*(0) ≥ *t*}. Here, for simplicity, we consider the SNCSTM of Section 3, where there are two time points.

When there is no censoring before time *t*, *P*{*T*(0) ≥ *t*} can be estimated for *t* ≤ *s*_1_ as the average over the *n* individuals of the adjusted survival indicator $I(T\geq t)\exp\left(A_{0}{\hat{\psi }}_{0(0)}t\right)$, and for *t > s*_1_ as the average of the adjusted survival indicator $I(T\geq t)\exp\left\{A_{0}{\hat{\psi }}_{0(0)}s_{1}+\left(A_{0}{\hat{\psi }}_{0(1)}+A_{1}{\hat{\psi }}_{1}\right)\left(t-s_{1}\right)\right\}$. If there is censoring before time *t*, *P*{*T*(0) ≥ *t*} can be estimated as the weighted average of the same adjusted indicators, excluding individuals who are censored before time *t*, and with the weights being one over the estimated probability of remaining uncensored at the earlier of times *t* and *T*, rather as in marginal structural Cox models.

See Appendix D for Data S1 for full details of how to estimate *P*{*T*(0) ≥ *t*} for the general SNCSTM and when there is censoring before time *t*.

## 7 Application to Uk Cystic Fibrosis Registry

SDKV used their Method 2 to estimate the causal effect of the drug DNase on survival of Cystic Fibrosis patients from data on 2386 adults with Cystic Fibrosis from the UK CF Registry.^21^ In this section we repeat their analysis but this time using the estimation method described in the current article, in order to demonstrate that it does indeed produce causal effect estimates that are very close to those of Method 2.

SDKV took an individual’s first visit during 2008 to 2015 as baseline visit and used data on this and up to eight follow-up visits. Median time between visits was 1.00 years (interquartile range 0.93 to 1.07). Individuals were “treated” if they had used DNase since the previous visit and “untreated” otherwise. Those treated at a visit prior to their baseline visit were excluded. Individuals who underwent a transplant were censored at the time of transplant. Likewise, individuals who were not seen for 18 months were censored at the end of the 18 months. The percentage of treated patients increased from 14% at baseline visit to 52% at visit 8, and most patients who began using DNase continued to use it. The death rates while treated and untreated were, respectively, 0.019 (74 deaths in 3930 person-years) and 0.0075 (63 deaths in 8450 person-years), and so the ratio of the probabilities of surviving one year was exp(−0.019)/ exp(−0.0075) = 0.989. However, this may be due to confounding: sicker patients being more likely to receive treatment.

Using Method 2, SDKV fitted Model (9) to estimate the causal effect of delaying initiation of treatment by 1 year. Recall that *ψ_k_*_(*l*)_ describes the causal effect of *A_k_*, the exposure measured at visit *k*, on the hazard between visits *l* and *l* + 1 (0 ≤ *k* ≤ *l* ≤ 8). SDKV (re)defined *A_k_* as *A_k_* = 0 (*A_k_* = 1) for those treated (untreated) at visit *k*, so that exp(*ψ_k_*_(*k*)_) represents the multiplicative causal effect of intervening to start treatment at visit *k* rather than visit *k* + 1 on the probability of surviving for at least one year after visit *k*, among patients who survive to, and are untreated at, visit *k*. More generally, $\left(\sum_{k+m-1}^{l=k}\psi_{k(l)}\right)$ is the effect on the probability of surviving at least *m* years after visit *k* if visits are exactly annual. SDKV constrained this effect to be the same for all 0 ≤ *k* ≤ 8 (see Appendix B3 of Data S1 for how to do this here). (Potential) confounders at visit *k* were baseline variables sex, age, and genotype class (low, high, not assigned), and time-varying variables FEV_1_%, body mass index, days of IV antibiotic use, and binary indicators for four infections (P. aeruginosa, S. aureus, B. cepacia complex, Aspergillus), CF-related diabetes, smoking, and use of other mucoactive treatments and oxygen therapy. The same variables (and treatment) were included in models for inverse probability of censoring weights.

**Figure 2:**
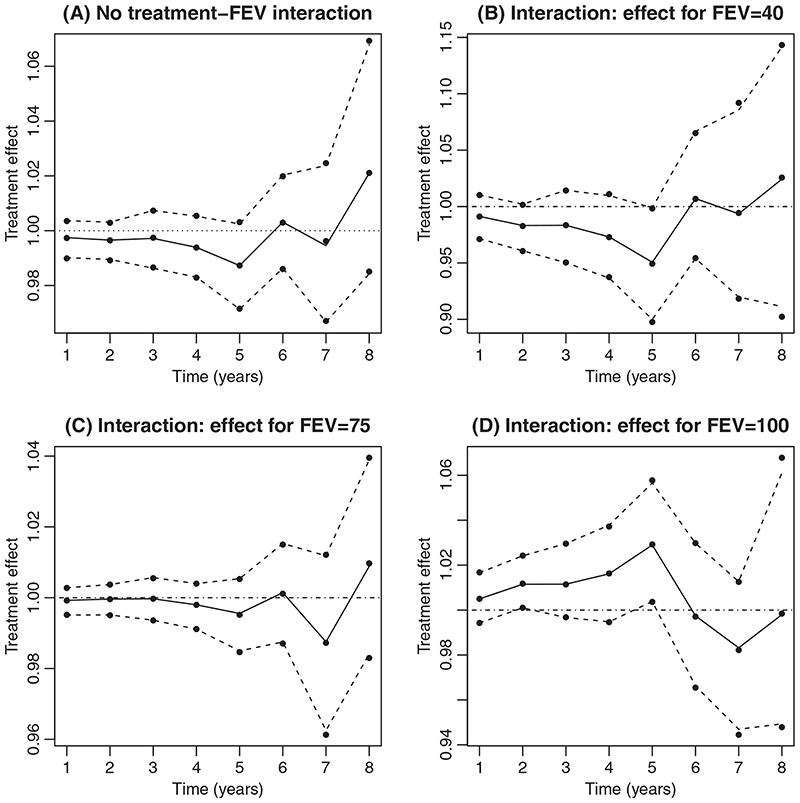
Ratio of the survival probabilities when treatment is initiated immediately compared to initiation being delayed by one year. A: from the model with no interaction. B, C and D: from the model with interaction between treatment and FEV_1_ *%*. Estimates from the method described in the current article are shown by solid lines, with 95% confidence limits shown by broken lines. Estimates and 95% confidence limits from Method 2 are shown by dots. A, model with no treatment-FEV interaction; B, effect for FEV = 40 in model with interaction; C, effect for FEV = 75 in model with interaction; D, effect for FEV = 100 in model with interaction

Figure 2A shows the estimates of $\left(\sum_{k+m-1}^{l=k}\psi_{k(l)}\right)$ obtained by SDKV. These suggest that starting treatment now rather than waiting may slightly decrease the survival probability, at least for the first five years. However, the confidence intervals (obtained by bootstrapping) include 1, that is, no treatment effect. Also shown are estimates we obtained using the method described in the present article. We see that this method closely approximates SDKV’s Method 2.

SDKV also fitted a SNCSTM with an interaction between treatment and the time-varying confounder FEV_1_%. Although the interaction was not significant, they presented the estimated ratios of survival probabilities for three value of FEV_1_%: 40, 75, and 100. Figure 2B to D shows these alongside the estimates we obtained. Again, these are very close.

**Figure 3:**
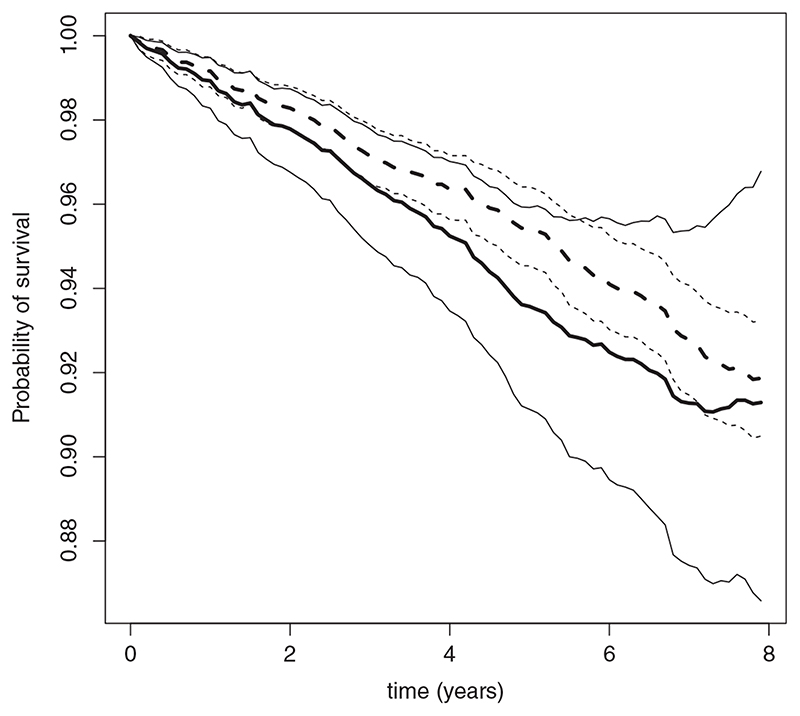
Estimated survival probability when treatment is begun at baseline, that is, *P*{*T*(0) ≥ *t*} (thick solid line), and estimated survival probability under the treatment regime observed in the cohort, that is, *P*(*T* ≥ *t*) (thick broken line), along with 95% confidence limits (thin solid lines for *P*{*T*(0) ≥ *t*} and thin broken lines for *P*(*T* ≥ *t*)).

Finally, Figure 3 shows the estimated survival probability when treatment is begun at baseline (ie, *P*{*T*(0) ≥ *t*}), using the SNCSTM of Figure 2A. This probability is less than the estimated probability under the treatment regime prevailing in the cohort (ie, *P*(*T* ≥ *t*)), but confidence intervals overlap considerably.

## 8 Discussion

The ability to use standard software has contributed greatly to the success of IPW methods for marginal structural Cox models, relative to other methods for time-varying confounding. The ability also to implement g-estimation for SNCSTMs in standard software, as described here, greatly increases the potential for uptake of this method.

In many settings, some exposure trajectories are implausible for certain individuals. Estimators that involve inverse weighting by probability of exposure trajectory can then be unstable. Instead, g-estimation of SNCSTMs may be particularly attractive in such case. SNCSTMs describe the effect of the next exposure conditional on the exposure and confounder histories. This offers the possibility of excluding those strata of the population that are composed of individuals whose next exposure is almost guaranteed by their histories to take one particular value (for a binary exposure) or to lie in a narrow range of values (for a continuous exposure). Even if such individuals are included, the form of the g-estimator is such that they make very little contribution to the exposure effect estimate (as it turns out to weight the observations by the difference between the observed and expected exposure). SNCSTMs make assumptions about how the exposure effect depends on the histories, which enables them to borrow information across strata, giving more weight to those strata that carry more information about the exposure effect (in particular, those strata in which the next exposure varies the most). The price paid for this ability to borrow information is potential bias when these assumptions are incorrect. In linear structural nested models, we have shown that inadvertently ignoring the possibility of effect modification by covariates need not be damaging, in that g-estimation then consistently estimates (optimal) weighted averages of the exposure effects across strata.^22^ The impact of ignoring such effect modification in SNCSTMs remains to be evaluated. With continuous exposures, a major advantage of g-estimation of SNCSTMs is that it relies solely on models for the exposure mean, thus overcoming the need for modelling, and inverse weighting by, the exposure density.

SNCSTMs imply multiplicative models for the probability (risk) of survival. This gives rise to causal effects that can be expressed as relative survival risks. These are more easily interpreted than hazard ratios, which are commonly reported when fitting marginal structural Cox models.^23^ As with other multiplicative models for risk, caution is warranted when survival risks are close to one, because the model does not constrain probabilities to stay below one. The SNCSTM models the effect only of exposures, not of confounders, on the survival probability, which may alleviate the impact of this lack of constraint. However, in future work, it will be interesting to exploit recent work on relative risk estimation by Richardson and colleagues^24,25^ to remove this concern entirely.

## Supplementary Material

Supplementary File

## Data Availability

This work used anonymized data from the UK Cystic Fibrosis Registry, which has Research Ethics Approval (REC ref: 07/Q0104/2). The use of the data was approved by the Registry Research Committee. Data are available following application to the Registry Research Committee. https://www.cysticfibrosis.org.uk/the-work-we-do/uk-cf-registry/apply-for-data-from-the-uk-cf-registry.
