# Supplementary material for: Using generalized linear models to implement g-estimation for survival data with time-varying confounding
Source: Stat Med. Author manuscript; Available in PMC 2022 Jan 5. (PMC7612171; doi:10.1002/sim.8997)
Supplement: Supplementary File [file EMS140638-supplement-Supplementary_File.pdf]

Using Generalised Linear Models to Implement G-estimation for Survival Data with Time-Varying Confounding

Seaman SR, Keogh RH, Dukes O and Vansteelandt S

Web Appendices

A The general model

Let K denote the number of post-baseline exposures. So, the exposures are $A_0, A_1, \ldots, A_K$. We denote the time at which A_k is measured on the individual as S_k ($k = 0, \ldots, K$). These exposure measurement times may be different for different individuals, although the baseline exposure is assumed to be measured at time zero on all individuals, and so $S_0 = 0$ for all individuals. Also, let S_{K+1} denote an administrative censoring time, common to all individuals. We assume that the post-baseline exposure measurement times $\bar{S} = (S_1, \ldots, S_K)$ are planned or randomly chosen at baseline using only baseline confounder information, i.e. L_0. When exposure measurement times are the same for all individuals, we can omit the term $\bar{S}$ in all the expressions below.

We assume that $T(\bar{A}_{k-1}, 0)$ is independent of A_k given $\bar{A}_{k-1}$, $\bar{L}_k$, $\bar{S}$ and $T \geq S_k$ for all $k = 0, \ldots, K$. Note that, throughout these Web Appendices, $T(\bar{A}_{-1}, 0)$ should be interpreted as $T(0)$.

To allow the (controlled direct) causal effect of A_k on the hazard during the interval between times S_l and S_{l+1} (for $k \leq l \leq K$) to be modified by the history $(\bar{A}_{k-1}, \bar{L}_k)$ of exposure and confounders at time S_k and/or by the exposure measurement times $\bar{S}$, let $Z^{\text{int}}_{k(l)}$ be any function of $\bar{A}_{k-1}$, $\bar{L}_k$ and $\bar{S}$ (the superscript 'int' stands for 'interaction'), and let $Z_{k(l)}$ be the vector $Z_{k(l)} = (1, Z^{\text{int}}_{k(l)})$. For example, if the effect of A_k during the time interval between S_l and S_{l+1} is modified by L_k, then $Z^{\text{int}}_{k(l)} = L_k$ and $Z_{k(l)}$ is the vector $Z_{k(l)} = (1, L_k)$. If the effect is modified by,

for example, both L_k and A_{k-1}, then $Z_{k(l)}^{\text{int}}$ is the vector $Z_{k(l)}^{\text{int}} = (L_k, A_{k-1})$ and $Z_{k(l)} = (1, L_k, A_{k-1})$. If there is no effect modification, just let $Z_{k(l)} = 1$.

The general SNCSTM assumes that

$$h_{T(A_{k-1},0)}(t \mid \bar{A}_k, \bar{L}_k, \bar{S}) = h_{T(A_k,0)}(t \mid \bar{A}_k, \bar{L}_k, \bar{S}) - A_k Z_{k(l)}^{\top} \psi_{k(l)} \tag{1}$$

when $S_l \leq t < S_{l+1}$, or equivalently

$$\frac{P\{T(\bar{A}_{k-1}, 0) \geq t \mid \bar{A}_k, \bar{L}_k, \bar{S}, T \geq S_k\}}{P\{T(\bar{A}_k, 0) \geq t \mid \bar{A}_k, \bar{L}_k, \bar{S}, T \geq S_k\}} = \exp\left\{ \sum_{j=k}^{l-1} A_k Z_{k(j)}^{\top} \psi_{k(j)} (S_{j+1} - S_j) + A_k Z_{k(l)}^{\top} \psi_{k(l)} (t - S_l) \right\} \tag{2}$$

when $S_l \leq t < S_{l+1}$. Note that if there is no effect modification, $Z_{k(l)}^{\top} \psi_{k(l)}$ is just $\psi_{k(l)}$. If instead, for example, $Z_{k(l)} = (1, L_k^{\top})^{\top}$, then $\psi_{k(l)} = (\psi_{k(l)}^{0}, \psi_{k(l)}^{L\top})^{\top}$ and $Z_{k(l)}^{\top} \psi_{k(l)} = \psi_{k(l)}^{0} + \psi_{k(l)}^{L\top} L_k$.

Web Appendix B describes how to fit the general SNCSTM.

B Fitting the general SNCSTM

B.1 The basic procedure

Here we describe how to estimate the parameter $\psi_{k(l)}$ $(0 \leq k \leq l \leq K)$ of the general SNCSTM described in Web Appendix A.

If the exposure measurement times $\bar{S}$ are the same for all individuals, we shall say that the 'measurements are regular'; if $\bar{S}$ differs between individuals, we shall say that the 'measurements are irregular'. We shall refer to a GLM with gamma distribution and log link function as a 'gamma GLM'.

Recall that S_{K+1} denotes the administrative censoring time common to all individuals. We use C to denote the individual's censoring time. So, $C \leq S_{K+1}$. In this web appendix only (i.e. only in Web Appendices B.1–B.3), we use T to denote the minimum of the individual's failure time (which we shall denote in this web appendix as $\tilde{T}$) and censoring time C. So, $T = \tilde{T}$ if $\tilde{T} \leq C$ and $T = C$ if $\tilde{T} > C$.

For each $k = 0, \ldots, K$, specify a GLM for A_k given $\bar{A}_{k-1}$, $\bar{L}_k$, $\bar{S}$ and $\tilde{T} \geq S_k$ with canonical link function. We shall refer to this GLM as 'Model $\mathcal{A}_k$'. For example, if A_k is continuous, Model $\mathcal{A}_k$ could be a linear regression model; if A_k is binary, Model $\mathcal{A}_k$ could be a logistic regression model.

The parameters should be estimated in the following order: first $\psi_{0(0)}, \psi_{1(1)}, \ldots, \psi_{K(K)}$; then $\psi_{0(1)}, \psi_{1(2)}, \ldots, \psi_{K-1(K)}$; then $\psi_{0(2)}, \psi_{1(3)}, \ldots, \psi_{K-2(K)}$; and so on; the last parameter to be estimated is $\psi_{0(K)}$. For each k and l such that $0 \leq k \leq l \leq K$, let $\hat{\psi}_{k(l)}$ denote the estimate of $\psi_{k(l)}$ that is obtained in the following way.

For each of the individuals with $T \geq S_l$ and for each value of $t = S_k, S_k + \delta, S_k + 2\delta, S_K + 3\delta, \ldots$ that satisfies $S_l \leq t \leq S_{l+1}$ and $t \leq T$, create a copy of the individual and set this copy's value of a new time variable Q equal to t. We call these copies of individuals 'pseudo-individuals'.

Fit Model $\mathcal{A}_k$, with $Z_{k(l)}(Q - S_k)$ included as an extra covariate (or covariates, if there is effect modification), to the pseudo-individuals using weights $w_k(Q)$, where

$$w_k(Q) = \exp\left[\sum_{j=k+1}^{l} A_j \left\{\sum_{m=j}^{l-1} (S_{m+1} - S_m) Z_{j(m)}^{\top} \hat{\psi}_{j(m)} + (Q - S_l) Z_{j(l)}^{\top} \hat{\psi}_{j(l)}\right\}\right] \quad (3)$$

when $S_l \leq Q \leq S_{l+1}$. Let $\hat{e}_k(Q)$ denote the fitted values thus obtained, and let $\hat{\Delta}_k(Q) = A_k - \hat{e}_k(Q)$.

If $l > k$ and either there is effect modification or the measurements are irregular, then additional extra covariates should be included when fitting this model. Specifically, if there is effect modification and the measurements are regular, the extra covariate (or covariates, if there is more than one effect modifier) is $Z_{k(l)}^{\text{int}}$. If the measurements are irregular, the extra covariates are $Z_{k(k)}(S_{k+1} - S_k), Z_{k(k+1)}(S_{k+2} - S_{k+1}), \ldots, Z_{k(l-1)}(S_l - S_{l-1})$.

Seaman et al. (2020) [1] showed that if $\mathcal{A}_k$ is correctly specified, then $\hat{e}_k(Q)$ is a consistent estimate of $E\{A_k \mid \bar{A}_{k-1}, \bar{L}_k, \bar{S}, T(A_k, 0) \geq Q\}$.

Now fit the gamma GLM with covariate (or covariates, if there is effect modification) $-Z_{k(l)}\hat{\Delta}_k(Q)\delta$, no intercept, and outcome variable $I(T \geq Q+\delta)\exp\left(\sum_{j=k+1}^{l} A_j Z_{j(l)}^{\top}\hat{\psi}_{j(l)}\delta\right)$ to the pseudo-individuals, using weights $w_k(Q)$ and excluding the pseudo-individuals that have either $Q+\delta > S_{l+1}$ or both $C < T$ and $Q+\delta > C$. (The former pseudo-individuals are excluded because we are only using data from the period between times S_l and S_{l+1}. The latter are excluded because the failure status at time $Q+\delta$ of these pseudo-individuals is unknown.) Let $\hat{\psi}_{k(l)}$ be the resulting estimate of the coefficient of $-Z_{k(l)}\hat{\Delta}_k(Q)\delta$.

When the measurements are regular and $S_l - S_k$ and $S_{l+1} - S_k$ are multiples of δ, we estimate $\psi_{k(l)}$ using exactly the procedure that has just been described. The method described in the section 'Estimating the joint effect of two exposures' of our article is the special case where $K = 1$ and there is no effect modification (and $\psi_{1(1)}$ is written as ψ_1). Note that when measurements are regular, it is always possible to choose the value for δ so that $S_l - S_k$ and $S_{l+1} - S_k$ are multiples of it.

B.2 Modification of procedure when exposure measurement times differ between individuals

When $S_l - S_k$ and/or $S_{l+1} - S_k$ are not always multiples of δ (in particular, this would be the case if the measurements were irregular), the procedure described in Web Appendix B.1 is still valid (i.e. it yields a consistent estimate of $\psi_{k(l)}$), but it may ignore information on some failures that occur shortly after time S_l or shortly before S_{l+1}. This loss of information could be reduced by choosing a very small value for δ, which would ensure that $S_l - S_k$ and $S_{l+1} - S_k$ were nearly multiples of δ. However, choosing a very small value for δ would lead to a very large number of pseudo-individuals being created, which would make fitting of the models very slow. So, we instead propose that two modifications of the procedure described in Web Appendix B.1 be made when $S_l - S_k$ or $S_{l+1} - S_k$ are not always multiples of δ. The first modification involves creating up to two extra pseudo-individuals

from each of the individuals still at risk at time S_k. The second modification is a small change to the covariate(s) used in the gamma GLM. Together, these two modifications ensure that no information on failures that occur shortly after time S_l or shortly before S_{l+1} is ignored.

The modified procedure is as follows.

Create the pseudo-individuals exactly as described above. Call this set of pseudo-individuals the 'original set of pseudo-individuals'. Now introduce a new variable D. This variable will represent the length of the time interval during which we shall consider each pseudo-individual to be at risk of failure, i.e. the interval between times Q and $Q + D$. For pseudo-individuals with $Q + \delta \leq S_{l+1}$, set $D = \delta$. For pseudo-individuals with $Q + \delta > S_{l+1}$, instead set $D = S_{l+1} - Q$. (This is because we are only using data from the period between times S_l and S_{l+1}, and so for these individuals we end their risk period early, at time S_{l+1} rather than time $Q + \delta$.) Now, for each of the individuals with $T \geq S_l$, create up to two extra copies of that individual as follows. First, let Q_{first} equal the smallest of $S_k, S_k + \delta, S_k + 2\delta, S_k + 3\delta, \ldots$ that is greater than or equal to S_l for that individual. This is the smallest value of Q among the pseudo-individuals already created from that individual, unless no such pseudo-individuals were created. Now, unless $Q_{\text{first}} = S_l$, create one extra copy of that individual, with $Q = S_l$ and $D = Q_{\text{first}} - S_l$. (This step will ensure that we shall use information on failures that occur between times S_l and Q_{first}.) Second, if the measurements are regular and if $T \geq S_{l+1}$ and $S_{l+1} - S_k$ is not a multiple of δ for that individual, then create one extra copy of that individual, with $Q = S_{l+1}$ and $D = 0$. (This step will ensure that when we fit Model $\mathcal{A}_k$ with $Z_{k(l)}(Q - S_k)$ as an extra covariate, we shall use information on failures that occur shortly before time S_{l+1}.) Finally, add these extra copies to the original set of pseudo-individuals and call the resulting set the 'supplemented set of pseudo-individuals'. This supplemented set is guaranteed to contain exactly one pseudo-individual with $Q = S_l$ from each individual with $T \geq S_l$ and, if the

measurements are regular, exactly one pseudo-individual with $Q = S_{l+1}$ from each individual with $T \geq S_{l+1}$.

Table 1 contains examples of the supplemented set of pseudo-individuals created by this process.

Fit Model $\mathcal{A}_k$, with $Z_{k(l)}(Q - S_k)$ included as extra covariate(s), to a set of pseudo-individuals using weights $w_k(Q)$. If the measurements are regular, use the supplemented set of pseudo-individuals. If the measurements are irregular, use the original set of pseudo-individuals. Let $\hat{e}_k(Q)$ denote the fitted values thus obtained and let $\hat{\Delta}_k(Q) = A_k - \hat{e}_k(Q)$. Note that fitted values $\hat{e}_k(Q)$ (and then $\hat{\Delta}_k(Q)$) should be obtained for the entire supplemented set of pseudo-individuals, even if this model has been fitted only to the original set of pseudo-individuals.

If $l > k$ and either there is effect modification or the measurements are irregular, then additional extra covariates should be included when fitting this model, exactly as described in Web Appendix B.1.

Finally, fit the gamma GLM with covariate(s) $-Z_{k(l)}\hat{\Delta}_k(Q)D$, no intercept, and outcome variable $I(T \geq Q + D) \exp\left(\sum_{j=k+1}^{l} A_j Z_{j(l)}^\top \hat{\psi}_{j(l)} D\right)$ to the supplemented set of pseudo-individuals using weights $w_k(Q) \times \delta/D$ and excluding any pseudo-individuals with either $D = 0$ or both $C < T$ and $Q + D > C$. To avoid possible problems of numerical instability, we actually use weights $w_k(Q) \times \delta/D^*$, where D^* is the minimum of D and a very small number (e.g. 10^{-6}). Set $\hat{\psi}_{k(l)}$ equal to the resulting estimate of the coefficient of $-Z_{k(l)}\hat{\Delta}_k(Q)D$.

B.3 Imposing the constraint that $\psi_{k(k+m)} = \psi_{k'(k'+m)}$ for all k, k', m

In some applications, it may be desirable to impose the constraint that $\psi_{k(k+m)} = \psi_{k'(k'+m)}$ for all k, k', m. This constraint implies the effect of exposure measured at one visit on the hazard m visits later is the same for all visits, i.e. that immediate

Measurements are regular, with $S_0 = 0$, $S_1 = 4$, $S_2 = 8$, $S_3 = 12$									
Individual 1: failed or censored after time 12									
Q	8	8.5	9	9.5	10	10.5	11	11.5	12
D	0.5	0.5	0.5	0.5	0.5	0.5	0.5	0.5	0
$\mathcal{A}_1$ uses	Y	Y	Y	Y	Y	Y	Y	Y	Y
gamma uses	Y	Y	Y	Y	Y	Y	Y	Y	N
Individual 2: failed at time 10.3									
Q	8	8.5	9	9.5	10				
D	0.5	0.5	0.5	0.5	0.5				
$\mathcal{A}_1$ uses	Y	Y	Y	Y	Y				
gamma uses	Y	Y	Y	Y	Y				
Individual 3: censored at time 10.3									
Q	8	8.5	9	9.5	10				
D	0.5	0.5	0.5	0.5	0.5				
$\mathcal{A}_1$ uses	Y	Y	Y	Y	Y				
gamma uses	Y	Y	Y	Y	N				
Individual 4: failed or censored before time 8									
No pseudo-individuals created									
Measurements are regular, with $S_0 = 0$, $S_1 = 3.1$, $S_2 = 6.3$, $S_3 = 8.4$									
Individual 5: failed or censored after time 8.4									
Q	6.6	7.1	7.6	8.1	6.3*	8.4*			
D	0.5	0.5	0.5	0.3	0.3	0			
$\mathcal{A}_1$ uses	Y	Y	Y	Y	Y	Y			
gamma uses	Y	Y	Y	Y	Y	N			
Measurements are irregular									
Individual 6: $S_0 = 0$, $S_1 = 3.1$, $S_2 = 8.9$, $S_3 = 11.2$, failed or censored after time 11.2									
Q	9.1	9.6	10.1	10.6	11.1	8.9*	11.2†		
D	0.5	0.5	0.5	0.5	0.1	0.2	0		
$\mathcal{A}_1$ uses	Y	Y	Y	Y	Y	N	–		
gamma uses	Y	Y	Y	Y	Y	Y	–		

Table 1: Illustrative example of the creation of pseudo-individuals. Here, $K = 3$, $\delta = 0.5$, the common administrative censoring time S_{K+1} equals 16, and we are estimating $\psi_{1(2)}$. Pseudo-individuals are shown for six individuals. '$\mathcal{A}_1$ uses' (Y=yes, N=no) means this pseudo-individual is used when fitting model $\mathcal{A}_1$ with the extra covariate $Q - S_1$. 'gamma uses' means this pseudo-individual is used when fitting the gamma GLM. $*$This is an 'extra' pseudo-individual who belongs to the supplemented set of pseudo-individuals, but not to the original set. When measurements are irregular, such 'extra' pseudo-individuals are not used when fitting Model $\mathcal{A}_1$ with extra covariate $Q - S_1$. †The pseudo-individual in red is not created, because measurements are irregular, but it would have been created had the measurements been regular.

and later effects of exposure do not depend on the time at which it is received. This reduces the number of parameters and, as Seaman et al. (2020) [1] demonstrated, increases the precision of their estimates.

The estimation procedure described above is easily modified to impose this constraint. In Web Appendices B.1 and B.2, a separate gamma GLM was fitted to estimate each of the $K+1$ parameters $\psi_{0(0)}, \psi_{1(1)}, \ldots, \psi_{K(K)}$, each time using a different set of pseudo-individuals. The covariate was $-Z_{k(k)}\hat{\Delta}_k(Q)\delta$ when estimating $\psi_{k(k)}$ ($k = 0, \ldots, K$) and the resulting estimated coefficient of this covariate was used as the estimate of $\psi_{k(k)}$. To impose the constraint that $\psi_{0(0)} = \psi_{1(1)} = \ldots = \psi_{K(K)}$, instead pool the $K+1$ sets of pseudo-individuals and fit a single gamma GLM with a single covariate to the resulting single dataset. For the kth of the $K+1$ sets of pseudo-individuals making up the pooled set ($k = 0, \ldots, K$), this single covariate equals $-Z_{k(k)}\hat{\Delta}_k(Q)\delta$. The resulting estimated coefficient of this covariate is taken to be the estimate of $\psi_{0(0)} = \psi_{1(1)} = \ldots = \psi_{K(K)}$. Likewise, when estimating $\psi_{0(m)} = \psi_{1(1+m)} = \ldots = \psi_{K-m(K)}$ for any $m > 0$, pool the $K+1-m$ sets of pseudo-individuals that were used in Web Appendices B.1 and B.2 to estimate each of $\psi_{0(m)}, \psi_{1(1+m)}, \ldots, \psi_{K-m(K)}$ separately, and fit a single gamma GLM to the resulting single dataset.

C Inverse probability of censoring weighting

In this web appendix, we describe how to use inverse probability of censoring weights to deal with censoring that depends on more than just baseline covariates L_0 and exposure measurement times $\bar{S}$. We do this in the context of the general SNCSTM, which is described in Web Appendix A. We shall describe the required modifications to the fitting procedure described in Web Appendix B.

We shall assume that the conditional hazard of censoring at time t given that the failure time T is at least t and given the actual failure time T and the histories of

the exposure and confounders up to time T and the exposure measurement times $\bar{S}$ depends on at most the histories of the exposure and confounders up to time t and $\bar{S}$. Let $\lambda(t \mid \bar{A}_{\lfloor t \rfloor}, \bar{L}_{\lfloor t \rfloor}, \bar{S})$ denote this conditional hazard of censoring at time t. Here, $\bar{A}_{\lfloor t \rfloor}$ and $\bar{L}_{\lfloor t \rfloor}$ denote the histories of the exposure and of the confounders, respectively, at time t. That is, $\bar{A}_{\lfloor t \rfloor} = A_0$ if $t < S_1$, $\bar{A}_{\lfloor t \rfloor} = (A_0, A_1)$ if $S_1 \leq t < S_2$, $\bar{A}_{\lfloor t \rfloor} = (A_0, A_1, A_2)$ if $S_2 \leq t < S_3$, etc., and analogously for $\bar{L}_{\lfloor t \rfloor}$.

For $k = 0, \ldots, K$, let

$$w_k^C(t) = \exp\left\{ \int_{S_k}^{t} \lambda(s, \bar{A}_{\lfloor s \rfloor}, \bar{L}_{\lfloor s \rfloor}, \bar{S}) \, ds \right\}$$

for $t \geq S_k$. This can be interpreted as one over the conditional probability of remaining uncensored at time t given the individual's histories of exposure and confounders up to time t and exposure measurement times $\bar{S}$ and given that the individual has not failed or been censored by time S_k, in a hypothetical world in which individuals remain at risk of censoring even after they have died.

Now, when fitting Model $\mathcal{A}_k$ with extra covariate $Z_{k(l)}(Q - S_k)$, each pseudo-individual should be (additionally) weighted by $w_k^C(Q)$. That is, instead of using the weights $w_k(Q)$ in Web Appendix B, use weights $w_k(Q) \times w_k^C(Q)$. Then, when fitting the gamma GLM with outcome variable $I(T \geq Q+D) \exp\left(\sum_{j=k+1}^{l} A_j Z_{j(l)}^\top \hat{\psi}_{j(l)} D \right)$, each pseudo-individual should be (additionally) weighted by $w_k^C(Q + D)$ if $T \geq Q + D$ or by $w_k^C(T)$ if both $T < Q + D$ and $T < C$. That is, instead of using weight $w_k(Q)$ in Web Appendix B, use weight $w_k(Q) \times w_k^C(Q + D)$ if $T \geq Q + D$ or $w_k(Q) \times w_k^C(T)$ if both $T < Q + D$ and $T < C$.

Usually, $\lambda(s, \bar{A}_{\lfloor s \rfloor}, \bar{L}_{\lfloor s \rfloor}, \bar{S})$ (and hence $w_k^C(t)$) is unknown and must be estimated from the data. To do this, specify a model for $\lambda(t, \bar{A}_{\lfloor t \rfloor}, \bar{L}_{\lfloor t \rfloor}, \bar{S})$. For simplicity, one could use a parametric proportional hazards model with piecewise constant baseline hazard between each pair of subsequent exposure measurement times S_k

and S_{k+1} and with time-dependent covariates A_k and L_k. This would mean that

$$w_k^C(t) = \exp\left\{\sum_{j=k}^{l-1} \lambda(s, A_j, L_j, \bar{S})\ (S_{j+1} - S_j) + \lambda(s, A_l, L_l, \bar{S})\ (t - S_l)\right\}$$

when $S_l \leq t < S_{l+1}$. This is what Seaman et al. (2020) [1] did when analysing the UK Cystic Fibrosis registry data described in the section 'Application to UK Cystic Fibrosis Registry' of our article, and is therefore what we did too. Alternatively, one could use, for example, a Cox regression model and Breslow estimator of the baseline cumulative hazard, or an Aalen additive hazards model. The chosen survival model is fitted to the data, treating censoring as the event of interest and treating failure as a censoring event.

More stable (i.e. less variable) inverse probability of censoring weights can be obtained by specifying, for each $k = 0, \ldots, K$, an additional model for the conditional hazard of censoring (at times $t \geq S_k$) given $\bar{A}_k$ and $\bar{L}_k$ (and $\bar{S}$). This differs from the previous model in that it conditions only on the histories of the exposure and confounders up to time S_k (and on $\bar{S}$), rather than on the full histories $\bar{A}_{\lfloor t \rfloor}$ and $\bar{L}_{\lfloor t \rfloor}$ up to time t (and on $\bar{S}$). Let $\lambda_k(s, \bar{A}_{k-1}, \bar{L}_k, \bar{S})$ (for $t > S_k$) denote this hazard, and let

$$w_k^S(t) = \exp\left\{\int_{S_k}^{t} \lambda_k(s, \bar{A}_{k-1}, \bar{L}_k, \bar{S})\ ds\right\}$$

This additional model is fitted to the data, again treating censoring as the event of interest and failure as a censoring event.

For simplicity, one could use a parametric proportional hazards model for $\lambda_k(s, \bar{A}_{k-1}, \bar{L}_k, \bar{S})$ with the (time-constant) covariates A_k and L_k and piecewise constant baseline hazard between each pair of subsequent exposure measurement times. If this model is used, then

$$w_k^S(t) = \exp\left\{\sum_{j=k}^{l-1} \lambda_k(s, A_{k-1}, L_k, \bar{S})\ (S_{j+1} - S_j) - \lambda_k(s, A_{k-1}, L_k, \bar{S})\ (t - S_l)\right\}$$

when $S_l \leq t < S_{l+1}$.

Now, when fitting Model $\mathcal{A}_k$ with extra covariate $Z_{k(l)}(Q - S_k)$, each pseudo-individual should be weighted by $w_k(Q) \times \frac{w_k^C(Q)}{w_k^S(Q)}$. Then, when fitting the gamma GLM, each pseudo-individual should be weighted by $w_k(Q) \times \frac{w_k^C(Q+D)}{w_k^S(Q+D)}$ if $T \geq Q+D$ or by $w_k(Q) \times \frac{w_k^C(T)}{w_k^S(Q+D)}$ if both $T < Q + D$ and $T < C$.

Note that misspecification of the model for $\lambda_k(s, \bar{A}_{k-1}, \bar{L}_k, \bar{S})$ does not affect the consistency of the estimator of $\psi_{k(l)}$.

If $\lambda(t, \bar{A}_{\lfloor t \rfloor}, \bar{L}_{\lfloor t \rfloor}, \bar{S}) = \lambda(t, L_0, \bar{S})$, i.e. the censoring hazard depends only on L_0 and $\bar{S}$, then

$$\frac{w_k^C(Q + D)}{w_k^S(Q + D)} = 1$$

and, when $Q \leq T < Q + D$,

$$\frac{w_k^C(T)}{w_k^S(Q + D)} = \exp\left\{ -\int_T^{Q+D} \lambda(s, L_0, \bar{S})\, ds \right\}.$$

If δ (and hence D) is small, then the difference between T and $Q + D$ will be small for pseudo-individuals with $Q \leq T < Q + D$, and so the weight $\frac{w_k^C(T)}{w_k^S(Q+D)}$ will be close to one and can be ignored. This confirms the claim in the section 'Censoring' of our paper that no censoring weights are needed when the censoring hazard depends only on L_0 (and $\bar{S}$).

D Estimating $P\{T(0) \geq t\}$

When there is no censoring before time t, we can estimate $P\{T(0) \geq t\}$ as $n^{-1} \sum_{i=1}^n J_i(t)$, where

$$J(t) = I(T \geq t) \times \exp\left[\sum_{k=0}^K \sum_{l=k}^K I(S_l \geq t) \left\{ \min(t, S_{l+1}) - S_l \right\} A_k Z_{k(l)}^\top \hat{\psi}_{k(l)} \right]. \quad (4)$$

Here, $I(S_l \geq t)$ equals 1 if $S_l \geq t$ and equals 0 otherwise, and $\min(t, S_{l+1})$ equals t if $t < S_{l+1}$ and equals S_{l+1} otherwise.

Note that, when $K = 1$ and $Z_{k(l)} = 1$, $J(t)$ reduces to

$$
\begin{aligned}
& I(T \geq t) \exp\left\{\min(t, S_1)\ A_0 \hat{\psi}_{0(0)} + I(S_1 \geq t)(t - S_1)(A_0 \hat{\psi}_{0(1)} + A_1 \hat{\psi}_{1(1)})\right\}, \\
= & \begin{cases} I(T \geq t) \exp(A_0 \hat{\psi}_{0(0)} t) & \text{if } t \leq S_1 \\ I(T \geq t) \exp\left\{A_0 \hat{\psi}_{0(0)} S_1 + (A_0 \hat{\psi}_{0(1)} + A_1 \hat{\psi}_{1(1)})(t - S_1)\right\} & \text{if } t > S_1, \end{cases}
\end{aligned}
$$

which is the formula given in the Section 'Estimating survival probability when both exposures are set to zero' of our paper.

Now suppose there is censoring before time t. Just as in Web Appendix C, we shall assume that the conditional hazard of censoring at time t given that the failure time T is at least t and given the actual failure time T and the histories of the exposure and confounders up to time T and the exposure measurement times $\bar{S}$ depends on at most the histories of the exposure and confounders up to time t and $\bar{S}$. Also just as in Web Appendix C, we shall denote this conditional hazard of censoring at time t as $\lambda(t \mid \bar{A}_{\lfloor t \rfloor}, \bar{L}_{\lfloor t \rfloor}, \bar{S})$ and write $w_0^C(t) = \exp\left\{\int_0^t \lambda(s \mid \bar{A}_{\lfloor s \rfloor}, \bar{L}_{\lfloor s \rfloor}, \bar{S})\, ds\right\}$. With this assumption, $P\{T(0) \geq t\}$ will be consistently estimated as

$$
\frac{\sum_{i=1}^n I(C_i \geq t \text{ or } T_i < C_i) \times w_{0i}^C\{\min(t, T_i)\} \times J_i(t)}{\sum_{i=1}^n I(C_i \geq t \text{ or } T_i < C_i) \times w_{0i}^C\{\min(t, T_i)\}}.
$$

If $\lambda(s \mid \bar{A}_{\lfloor t \rfloor}, \bar{L}_{\lfloor t \rfloor}, \bar{S})$ depends neither on the histories of exposure and confounders nor on $\bar{S}$, we can estimate $w_{0i}^C(t)$ by using $\exp\{H_{\text{NA}}(t)\}$, where $H_{\text{NA}}(t)$ is the Nelson-Aalen estimator of the cumulative hazard, or alternatively by using the Kaplan-Meier estimator. When calculating this Nelson-Aalen or Kaplan-Meier estimator, censoring should be treated as the event of interest and failure should be treated as a censoring event.

More generally, one could estimate $\lambda(t \mid \bar{A}_{\lfloor t \rfloor}, \bar{L}_{\lfloor t \rfloor}, \bar{S})$, and hence $w_0^C(t)$, using a Cox regression model and the Breslow estimator of the baseline cumulative hazard. This is what we did for the analysis of the Cystic Fibrosis data described in the section 'Application to UK Cystic Fibrosis Registry' of our article.

E Proof of consistency of $\hat{\psi}_{k(l)}$

Here we provide a proof of consistency of the estimator of the parameter $\psi_{k(l)}$ ($0 \leq k \leq l \leq K$) in the general model described in Web Appendix A. The method used to calculate this estimator is described in Web Appendix B.

Let $R(t) = I(T \geq t)$. Let u and t be such that $S_l \leq u < t \leq S_{l+1}$. Write

$$\Delta_k(u, \bar{A}_{k-1}, \bar{L}_k, \bar{S}) = A_k - E\{A_k \mid \bar{A}_{k-1}, \bar{L}_k, \bar{S}, T(\bar{A}_k, 0) \geq u\}.$$

Define

$$\begin{aligned} & M_{k(l)}(u, t, \bar{A}_l, \bar{L}_l, \bar{S}, T) \\ & \quad = R(u)\, \Delta_k(u)\, w_k(u) \\ & \qquad \times \left[R(t) \exp\left\{ \sum_{j=k+1}^{l} A_j \psi_{j(l)}^{\top} Z_{j(l)}(t-u) + A_k \psi_{k(l)}^{\top} Z_{k(l)}(t-u) \right\} - 1 \right]. \end{aligned} \tag{5}$$

Let $\mathcal{B}_{k(l),i}$ denote the set of pairs of values of (Q, D) of copies created from individual i when estimating $\psi_{k(l)}$. For these, $S_l \leq Q \leq Q + D \leq S_{l+1}$.

Fitting the gamma GLM described in Web Appendix B involves solving estimating equations

$$\sum_{i=1}^{n} \sum_{\{(Q,D) \in \mathcal{B}_{k(l),i}\}} M_{k(l)}(Q, Q+D, \bar{A}_{li}, \bar{L}_{li}, \bar{S}_i, T_i) = 0, \tag{6}$$

with $E\{A_k \mid \bar{A}_{k-1}, \bar{L}_k, \bar{S}, T(\bar{A}_k, 0) \geq u\}$ in the $\Delta_k(u)$ term replaced by an estimate that Seaman et al. (2020) [1] showed is consistent, and with $\psi_{k+1(l)}, \ldots, \psi_{l(l)}$ replaced by previously obtained estimates. We shall argue by induction, and so we assume that these previously obtained estimates are consistent. Note that $M_{k(l)}(Q, Q+D, \bar{A}_l, \bar{L}_l, \bar{S}, T) = 0$ for copies that, because they have $Q > T$, are discarded and do not become pseudo-individuals, and for copies with $D = 0$.

We now prove that expression (5) has expectation zero given $\bar{A}_{k-1}$, $\bar{L}_k$, $\bar{S}$ and $T \geq S_k$ when $S_l \leq u < t \leq S_{l+1}$, and hence (6) are unbiased estimating equations for $\psi_{k(l)}$.

To simplify the notation slightly, we shall condition implicitly (rather than explicitly) on $\bar{S}$, and write $M_{k(l)}(u, t, \bar{A}_l, \bar{L}_l, \bar{S}, T)$ and $\Delta_k(u)$ just as M and Δ_k. Also, we define, for $k \le x \le l$ and $S_l \le u < t \le S_{l+1}$,

$$\begin{aligned}
\Pi_{kx} &\equiv \Pi_x(Q, \bar{A}_x, \bar{L}_x) \equiv \exp\left[\sum_{j=k+1}^{x} A_j \sum_{m=j}^{l} Z_{j(m)}^{\top} \psi_{j(m)} \{(S_{m+1} \wedge u) - S_m\}\right] \\
&\qquad\qquad\qquad\qquad\qquad\qquad\qquad\qquad\qquad\qquad \text{(for } x > k\text{)} \\
\Pi_{kk} &\equiv 1 \\
\Omega_{kl} &\equiv \Omega_l(u, t, \bar{A}_l, \bar{L}_l) \equiv \exp\left\{\sum_{j=k}^{l} A_j Z_{j(l)}^{\top} \psi_{j(l)}(t - u)\right\}.
\end{aligned}$$

To simplify notation, we shall write Π_{kx} and Ω_{kl} as just Π_x and Ω_l.

We can now write M as

$$M = \Delta_k \; R(u) \; \Pi_l \; \{R(t) \; \Omega_l - 1\}.$$

Now,

$$\begin{aligned}
&E\{M \mid \bar{A}_l, \bar{L}_l, R(u)\} \\
&= \Delta_k \; R(u) \; \Pi_l \times \{P(T \ge t \mid \bar{A}_l, \bar{L}_l, T \ge u) \; \Omega_l - 1\} \\
&= \Delta_k \; R(u) \; \Pi_l \times \left\{\frac{P(T \ge t \mid \bar{A}_l, \bar{L}_l, T \ge S_l)}{P(T \ge u \mid \bar{A}_l, \bar{L}_l, T \ge S_l)} \; \Omega_l - 1\right\} \\
&= \Delta_k \; R(u) \; \Pi_l \\
&\quad \times \left[\frac{P\{T(\bar{A}_{l-1}, 0) \ge t \mid \bar{A}_l, \bar{L}_l, T \ge S_l\} \exp\{-A_l Z_{l(l)}^{\top} \psi_{l(l)}(t - S_l)\}}{P\{T(\bar{A}_{l-1}, 0) \ge u \mid \bar{A}_l, \bar{L}_l, T \ge S_l\} \exp\{-A_l Z_{l(l)}^{\top} \psi_{l(l)}(u - S_l)\}} \; \Omega_l - 1\right]
\end{aligned} \quad (7)$$

$$\begin{aligned}
&= \Delta_k \; R(u) \; \Pi_l \\
&\quad \times \left[\frac{P\{T(\bar{A}_{l-1}, 0) \ge t \mid \bar{A}_l, \bar{L}_l, T \ge S_l\}}{P\{T(\bar{A}_{l-1}, 0) \ge u \mid \bar{A}_l, \bar{L}_l, T \ge S_l\}} \; \Omega_{l-1} - 1\right] \\
&= \Delta_k \; R(u) \; \Pi_l \times [P\{T(\bar{A}_{l-1}, 0) \ge t \mid \bar{A}_l, \bar{L}_l, T(\bar{A}_{l-1}, 0) \ge u\} \; \Omega_{l-1} - 1].
\end{aligned} \quad (8)$$

Note that line (7) follows from the SNCSTM. Hence,

$$
\begin{aligned}
&E\{M \mid \bar{A}_l, \bar{L}_l, R(S_l)\} \\
&= \Delta_k\ R(S_l) \times P(T \geq u \mid \bar{A}_l, \bar{L}_l, T \geq S_l)\ \Pi_l \\
&\quad \times [P\{T(\bar{A}_{l-1}, 0) \geq t \mid \bar{A}_l, \bar{L}_l, T(\bar{A}_{l-1}, 0) \geq u\}\ \Omega_{l-1} - 1] \\
&= \Delta_k\ R(S_l) \times P\{T(\bar{A}_{l-1}, 0) \geq u \mid \bar{A}_l, \bar{L}_l, T \geq S_l\} \exp\{-A_l Z_{l(l)}^\top \psi_{l(l)}(u - S_l)\}\ \Pi_l \\
&\quad \times [P\{T(\bar{A}_{l-1}, 0) \geq t \mid \bar{A}_l, \bar{L}_l, T(\bar{A}_{l-1}, 0) \geq u\}\ \Omega_{l-1} - 1] \qquad (9) \\
&= \Delta_k\ R(S_l) \times P\{T(\bar{A}_{l-1}, 0) \geq u \mid \bar{A}_l, \bar{L}_l, T \geq S_l\}\ \Pi_{l-1} \\
&\quad \times [P\{T(\bar{A}_{l-1}, 0) \geq t \mid \bar{A}_l, \bar{L}_l, T(\bar{A}_{l-1}, 0) \geq u\}\ \Omega_{l-1} - 1] \qquad (10) \\
&= \Delta_k\ R(S_l)\ \Pi_{l-1} \times [P\{T(\bar{A}_{l-1}, 0) \geq t \mid \bar{A}_l, \bar{L}_l, T \geq S_l\}\ \Omega_{l-1} \\
&\qquad\qquad - P\{T(\bar{A}_{l-1}, 0) \geq u \mid \bar{A}_l, \bar{L}_l, T \geq S_l\} \times 1] \qquad (11)
\end{aligned}
$$

Note that line (9) follows from the SNCSTM. Hence,

$$
\begin{aligned}
&E\{M \mid \bar{A}_{l-1}, \bar{L}_{l-1}, R(S_l)\} \\
&= \Delta_k\ R(S_l)\ \Pi_{l-1} \times [P\{T(\bar{A}_{l-1}, 0) \geq t \mid \bar{A}_{l-1}, \bar{L}_{l-1}, T \geq S_l\}\ \Omega_{l-1} \\
&\qquad\qquad - P\{T(\bar{A}_{l-1}, 0) \geq u \mid \bar{A}_{l-1}, \bar{L}_{l-1}, T \geq S_l\}]
\end{aligned}
$$

It then follows that

$$
\begin{aligned}
&E\{M \mid \bar{A}_{l-1}, \bar{L}_{l-1}, R(S_{l-1})\} \\
&= \Delta_k\ R(S_{l-1}) \times P(T \geq S_l \mid \bar{A}_{l-1}, \bar{L}_{l-1}, T \geq S_{l-1})\ \Pi_{l-1} \\
&\quad \times [P\{T(\bar{A}_{l-1}, 0) \geq t \mid \bar{A}_{l-1}, \bar{L}_{l-1}, T \geq S_l\}\ \Omega_{l-1} \\
&\qquad -P\{T(\bar{A}_{l-1}, 0) \geq u \mid \bar{A}_{l-1}, \bar{L}_{l-1}, T \geq S_l\}] \\
&= \Delta_k\ R(S_{l-1})\ \Pi_{l-1} \\
&\quad \times [P\{T(\bar{A}_{l-1}, 0) \geq t \mid \bar{A}_{l-1}, \bar{L}_{l-1}, T \geq S_{l-1}\}\ \Omega_{l-1} \\
&\qquad -P\{T(\bar{A}_{l-1}, 0) \geq u \mid \bar{A}_{l-1}, \bar{L}_{l-1}, T \geq S_{l-1}\}] \\
&= \Delta_k\ R(S_{l-1})\ \Pi_{l-1} \times P\{T(\bar{A}_{l-1}, 0) \geq u \mid \bar{A}_{l-1}, \bar{L}_{l-1}, T \geq S_{l-1}\} \\
&\quad \times [P\{T(\bar{A}_{l-1}, 0) \geq t \mid \bar{A}_{l-1}, \bar{L}_{l-1}, T(\bar{A}_{l-1}, 0) \geq u\}\ \Omega_{l-1} - 1] \\
&= \Delta_k\ R(S_{l-1})\ \Pi_{l-1} \times P\{T(\bar{A}_{l-1}, 0) \geq u \mid \bar{A}_{l-1}, \bar{L}_{l-1}, T \geq S_{l-1}\} \\
&\quad \times [P\{T(\bar{A}_{l-2}, 0) \geq t \mid \bar{A}_{l-1}, \bar{L}_{l-1}, T(\bar{A}_{l-2}, 0) \geq u\}\ \Omega_{l-2} - 1] \qquad (12) \\
&= \Delta_k\ R(S_{l-1})\ \Pi_{l-2} \times P\{T(\bar{A}_{l-2}, 0) \geq u \mid \bar{A}_{l-1}, \bar{L}_{l-1}, T \geq S_{l-1}\} \\
&\quad \times [P\{T(\bar{A}_{l-2}, 0) \geq t \mid \bar{A}_{l-1}, \bar{L}_{l-1}, T(\bar{A}_{l-2}, 0) \geq u\}\ \Omega_{l-2} - 1] \qquad (13) \\
&= \Delta_k\ R(S_{l-1})\ \Pi_{l-2} \times [P\{T(\bar{A}_{l-2}, 0) \geq t \mid \bar{A}_{l-1}, \bar{L}_{l-1}, T \geq S_{l-1}\}\ \Omega_{l-2} \\
&\qquad\qquad -P\{T(\bar{A}_{l-2}, 0) \geq u \mid \bar{A}_{l-1}, \bar{L}_{l-1}, T \geq S_{l-1}\}] \qquad (14)
\end{aligned}
$$

Note that line (12) follows from the SNCSTM, using the same argument that led to line (8). Likewise, line (13) follows from the SNCSTM, using the same argument that led to line (10).

From lines (11) and (14), we see that, by using induction, we can arrive at

$$
\begin{aligned}
&E\{M \mid \bar{A}_{k+1}, \bar{L}_{k+1}, R(S_{k+1})\} \\
&= \Delta_k\ R(S_{k+1}) \times [P\{T(\bar{A}_k, 0) \geq t \mid \bar{A}_{k+1}, \bar{L}_{k+1}, T \geq S_{k+1}\}\ \Omega_k \\
&\qquad\qquad -P\{T(\bar{A}_k, 0) \geq u \mid \bar{A}_{k+1}, \bar{L}_{k+1}, T \geq S_{k+1}\}] \\
&= \Delta_k\ R(S_{k+1}) \times [P\{T(\bar{A}_k, 0) \geq t \mid \bar{A}_{k+1}, \bar{L}_{k+1}, T \geq S_{k+1}\} \\
&\quad \times \exp\{A_k Z_{k(l)}^\top \psi_{k(l)}(t-u)\} - P\{T(\bar{A}_k, 0) \geq u \mid \bar{A}_{k+1}, \bar{L}_{k+1}, T \geq S_{k+1}\}]
\end{aligned}
$$

It then follows that

$$
\begin{aligned}
& E\{M \mid \bar{A}_k, \bar{L}_k, R(S_{k+1})\} \\
& = \Delta_k \; R(S_{k+1}) \times [P\{T(\bar{A}_k, 0) \geq t \mid \bar{A}_k, \bar{L}_k, T \geq S_{k+1}\} \\
& \quad \times \exp\{A_k Z_{k(l)}^{\top} \psi_{k(l)}(t-u)\} - P\{T(\bar{A}_k, 0) \geq u \mid \bar{A}_k, \bar{L}_k, T \geq S_{k+1}\}]
\end{aligned}
$$

Hence,

$$
\begin{aligned}
& E\{M \mid \bar{A}_k, \bar{L}_k, R(S_k)\} \\
& = \Delta_k \; R(S_k) \times P(T \geq S_{k+1} \mid \bar{A}_k, \bar{L}_k, T \geq S_k) \\
& \quad \times [P\{T(\bar{A}_k, 0) \geq t \mid \bar{A}_k, \bar{L}_k, T \geq S_{k+1}\} \; \exp\{A_k Z_{k(l)}^{\top} \psi_{k(l)}(t-u)\} \\
& \quad\quad - P\{T(\bar{A}_k, 0) \geq u \mid \bar{A}_k, \bar{L}_k, T \geq S_{k+1}\}] \\
& = \Delta_k \; R(S_k) \times [P\{T(\bar{A}_k, 0) \geq t \mid \bar{A}_k, \bar{L}_k, T \geq S_k\} \; \exp\{A_k Z_{k(l)}^{\top} \psi_{k(l)}(t-u)\} \\
& \qquad\qquad - P\{T(\bar{A}_k, 0) \geq u \mid \bar{A}_k, \bar{L}_k, T \geq S_k\}] \\
& = \Delta_k \; R(S_k) \times P(T(\bar{A}_k, 0) \geq u \mid \bar{A}_k, \bar{L}_k, T \geq S_k) \\
& \quad \times [P\{T(\bar{A}_k, 0) \geq t \mid \bar{A}_k, \bar{L}_k, T(\bar{A}_k, 0) \geq u\} \; \exp\{A_k Z_{k(l)}^{\top} \psi_{k(l)}(t-u)\} - 1] \\
& = \Delta_k \; R(S_k) \times P(T(\bar{A}_k, 0) \geq u \mid \bar{A}_k, \bar{L}_k, T \geq S_k) \\
& \quad \times [P\{T(\bar{A}_{k-1}, 0) \geq t \mid \bar{A}_k, \bar{L}_k, T(\bar{A}_{k-1}, 0) \geq u\} - 1] \qquad (15) \\
& = \Delta_k \; R(S_k) \times P(T(\bar{A}_k, 0) \geq u \mid \bar{A}_k, \bar{L}_k, T \geq S_k) \\
& \quad \times [P\{T(\bar{A}_{k-1}, 0) \geq t \mid \bar{A}_{k-1}, \bar{L}_k, T(\bar{A}_{k-1}, 0) \geq u\} - 1] \qquad (16) \\
& = \Delta_k \; R(S_k) \times P(T(\bar{A}_k, 0) \geq u \mid \bar{A}_k, \bar{L}_k, T \geq S_k) \times d(\bar{A}_{k-1}, \bar{L}_k)
\end{aligned}
$$

where $d(\bar{A}_{k-1}, \bar{L}_k) = P\{T(\bar{A}_{k-1}, 0) \geq t \mid \bar{A}_{k-1}, \bar{L}_k, T(\bar{A}_{k-1}, 0) \geq u\} - 1$. Note that line (15) follows from the SNCSTM, using the same argument that led to line (10), and line (16) follows from the assumption that $T(\bar{A}_{k-1}, 0) \perp\!\!\!\perp A_k \mid \bar{A}_{k-1}, \bar{L}_k, T \geq S_k$.

So, we can write

$$
\begin{aligned}
&E\{M \mid \bar{A}_k, \bar{L}_k, T \geq S_k\} \\
&= [A_k - E\{A_k \mid \bar{A}_{k-1}, \bar{L}_k, T(\bar{A}_k, 0) \geq u\}] \times P(T(\bar{A}_k, 0) \geq u \mid \bar{A}_k, \bar{L}_k, T \geq S_k) \\
&\quad \times d(\bar{A}_{k-1}, \bar{L}_k) \\
&= E\big([A_k - E\{A_k \mid \bar{A}_{k-1}, \bar{L}_k, T(\bar{A}_k, 0) \geq u\}] \, R_{(\bar{A}_k,0)}(u) \mid \bar{A}_k, \bar{L}_k, T \geq S_k\big) \\
&\quad \times d(\bar{A}_{k-1}, \bar{L}_k)
\end{aligned}
$$

It now follows that

$$
\begin{aligned}
&E\{M \mid \bar{A}_{k-1}, \bar{L}_k, T \geq S_k\} \\
&= E\big([A_k - E\{A_k \mid \bar{A}_{k-1}, \bar{L}_k, T(\bar{A}_k, 0) \geq u\}] \, R_{(\bar{A}_k,0)}(u) \mid \bar{A}_{k-1}, \bar{L}_k, T \geq S_k\big) \\
&\quad \times d(\bar{A}_{k-1}, \bar{L}_k) \\
&= E\big([A_k - E\{A_k \mid \bar{A}_{k-1}, \bar{L}_k, T(\bar{A}_k, 0) \geq u\}] \mid \bar{A}_{k-1}, \bar{L}_k, T(\bar{A}_k, 0) \geq u\big) \\
&\quad \times P\{T(\bar{A}_k, 0) \geq u \mid \bar{A}_{k-1}, \bar{L}_k, T \geq S_k\} \times d(\bar{A}_{k-1}, \bar{L}_k) \\
&= 0 \times P\{T(\bar{A}_k, 0) \geq u \mid \bar{A}_{k-1}, \bar{L}_k, T \geq S_k\} \times d(\bar{A}_{k-1}, \bar{L}_k) \\
&= 0
\end{aligned}
$$

as required.

F Relation between $\hat{\psi}$ and 'Method 2'

In this section, we show that the causal effect estimators described in Sections 2, 3 and 5 are closely related to those obtained from Seaman et al.'s (2020)[1] Method 2. For simplicity, we consider the situation of a point exposure A, with no effect modification and only administrative censoring, just as in Section 2. However, the proof below can be generalised to show that the same relation between the estimators exists when there are multiple exposures $A_0, \ldots, A_K$, effect modification and random censoring.

The estimator $\hat{\psi}$ of the causal effect of a point exposure, described in Section 2, was motivated by the equation

$$\frac{P\{T(0) \geq t+\delta \mid A, L, T(0) \geq t\}}{P\{T \geq t+\delta \mid A, L, T \geq t\}} = \exp(A\psi\delta). \tag{17}$$

Seaman et al.'s motivation for their Method 2 was very similar, but with δ taken to be infinitely small. Write $\Delta(L,t) = A - E(A \mid L, T \geq t)$. Just as the estimating equation for $\tilde{\psi}$ is

$$\sum_{i=1}^{n} \Delta(L_i, 0) \times \left[I(T_i \geq t) \exp\{\tilde{\psi}\Delta(L_i,0)t\} - 1\right] = 0$$

with $\Delta(L,0)$ replaced by the estimate $A - \hat{e}(L,0)$, the estimating equation for $\hat{\psi}$ is the sum over the n individuals of

$$\sum_{j=1}^{\infty} I\{T \geq \delta(j-1)\} \times \Delta\{L, \delta(j-1)\} \times \delta \times \big(I(T \geq \delta j) \exp\left[\psi\Delta\{L, \delta(j-1)\}\delta\right] - 1\big) = 0, \tag{18}$$

with $\Delta\{L, \delta(j-1)\}$ replaced by the estimate $A - \hat{e}\{L, \delta(j-1)\}$. Equation (18) is equivalent to

$$\begin{aligned} &\delta \sum_{j=1}^{\infty} \Delta\{L, \delta(j-1)\} \times I(T \geq \delta j) \times \big(\exp\left[\psi\Delta\{L, \delta(j-1)\}\delta\right] - 1\big) \\ &-\delta \sum_{j=1}^{\infty} \Delta\{L, \delta(j-1)\} \times I\{T \geq \delta(j-1)\} \times \{1 - (T \geq \delta j)\} = 0. \end{aligned} \tag{19}$$

Dividing both sides by $-\delta$ and then using the fact that $\lim_{\delta \to 0}\{\exp(\delta) - 1\}/\delta = 1$, we see that as $\delta \to 0$ equation (19) becomes

$$-\int_0^{\infty} I(T \geq u)\, \Delta(L,u)\, \psi\, \Delta(L,u)\, du + dN(T)\Delta(L,T) = 0,$$

which is equivalent to

$$\int_0^{\infty} I(T \geq u)\, \Delta(L,u)\, \{dN(u) - \psi\, \Delta(L,u)\}\, du = 0. \tag{20}$$

Equation (20) is the same as the estimating equation of Method 2 of Seaman et al., except that in Method 2 the term $dN(u) - \psi\Delta(L,u)$ is replaced by $dN(u) -$

$\psi\Delta(L,0)$. Unless ψ is large, $\Delta(L,u)$ will be close to $\Delta(L,0)$. The quantities $\Delta(L,u)$ and $\Delta(L,0)$ are unknown and must be replaced by estimates. Both the method described in this article and Method 2 estimate $\Delta(L,u)$ and $\Delta(L,0)$ in exactly the same way, by fitting a canonical GLM for $E(A \mid L)$.

In Web Appendix B, when the exposure measurement times differ between individuals we introduced extra weights δ/D when fitting the gamma GLM. We did this so that the contribution of a pseudo-individual to the estimating function becomes

$$\begin{aligned}
&\frac{\delta}{D} \times \Big(D\Delta(Q) \times I(T \geq Q+D) \times \big[\exp\{\psi\Delta(Q)D\} - 1\big] \\
&\quad -D\Delta(Q) \times I(T \geq Q) \times \{1 - I(T \geq Q+D)\}\Big) \\
&= \delta \times \big(\Delta(Q) \times I(T \geq Q+D) \times \big[\exp\{\psi\Delta(Q)D\} - 1\big] \\
&\quad -\Delta(Q) \times I(T \geq Q) \times \{1 - I(T \geq Q+D)\}\big)
\end{aligned}$$

Without the weights δ/D, the failure of a pseudo-individual with $D = \delta$ during the time interval $[Q, Q+D]$ would be given more weight than the failure of a pseudo-individual with $D < \delta$. By weighting by δ/D, all failures are given the same weight.

G Example R code for fitting SNCSTM

The files 'example_regular.r' and 'example_irregular.r' contain examples of R code for fitting the SNCSTM. For these examples, a dataset is randomly generated in the following way.

There are $K + 1 = 4$ exposure measurement times and two time-dependent confounders (i.e. each L_k consists of two variables). These confounders and the exposure were generated as: $L_0 \sim N\left((0,0),\ \Sigma\right)$, $A_0 \sim N(3 + (0.2, 0.1)^\top L_0,\ 0.9^2)$, $L_k \sim N\left(\Omega L_{k-1} + (0.1, 0.05)^\top A_{k-1},\ \Sigma\right)$ and $A_k \sim N(3 + (0.1, 0.05)^\top L_k,\ 0.7^2)$ $(k \geq 1)$, where $\Sigma = \begin{bmatrix} 0.5 & 0.2 \\ 0.2 & 0.5 \end{bmatrix}$ and $\Omega = \begin{bmatrix} 0.2 & 0.2 \\ 0.1 & 0.1 \end{bmatrix}$. The hazard of failure during the interval between the kth and $(k+1)$th exposure measurement times

was $0.34 + (0.03, 0.03)^\top L_k - 0.04A_k - 0.0145A_{k-1}I(k \geq 1) - 0.0055A_{k-2}I(k \geq 2) - 0.00245A_{k-3}I(k = 3)$.

For this data-generating mechanism, there is no effect modification and the true exposure effects are $\psi_{k(k)} = -0.04$ (for $k = 0, 1, 2, 3$), $\psi_{k(k+1)} = -0.01$ (for $k = 0, 1, 2$), $\psi_{k(k+2)} = -0.004$ (for $k = 0, 1$) and $\psi_{0(3)} = -0.002$.

We considered both a scenario in which the exposure measurement times are the same for all individuals ('measurements are regular') and where they differ ('measurements are irregular'). When measurements were regulars, $S_{ik} = k$. When measurements were irregular, inter-measurement times $S_{k+1,i} - S_{ki}$ were independently uniformly distributed on $[0.5, 1.5]$.

Censoring was imposed by generating a censoring time completely at random from an exponential distribution with rate of 0.2. Individuals who had still not failed or been censored by time 4 were administratively censored at that time.

When $n = 1000$, the number of individuals observed to fail between time $S_0 = 0$ and S_1, between $S_1 = 1$ and S_2, between $S_2 = 1$ and S_3, and between S_3 and time 4 are approximately 200, 100, 50 and 50, respectively. The corresponding numbers of individuals who are censored were approximately 140, 100, 70 and 50.

We also considered two scenarios (one with regular and one with irregular measurements) in which there is effect modification. In these scenarios, the dataset was generated in the same way as described above, except that the causal effect of A_k on the hazard depended on L_{k1}, the first element of L_k. For this data-generating mechanism,

$$h_{T(A_{k-1},0)}(t \mid \bar{A}_k, \bar{L}_k) = h_{T(A_k,0)}(t \mid \bar{A}_k, \bar{L}_k) - A_k(1, L_{k1})^\top \psi_{k(l)}$$

when $s_l \le t < s_{l+1}$, or equivalently,

$$\frac{P\{T(\bar{A}_{k-1},0) \ge t \mid \bar{A}_k, \bar{L}_k, T \ge s_k\}}{P\{T(\bar{A}_k,0) \ge t \mid \bar{A}_k, \bar{L}_k, T \ge s_k\}}$$
$$= \exp\left\{\sum_{j=k}^{l-1} A_k(1,L_{k1})^\top \psi_{k(j)}(s_{j+1}-s_j) + A_k(1,L_{k1})^\top \psi_{k(l)}(t-s_l)\right\}$$

when $s_l \le t < s_{l+1}$. The true exposure effects are $\psi_{k(k)} = (-0.04, 0.004)$ (for $k = 0,1,2,3$), $\psi_{k(k+1)} = (-0.01, 0.002)$ (for $k = 0,1,2$), $\psi_{k(k+2)} = (-0.004, 0)$ (for $k = 0,1$) and $\psi_{0(3)} = (-0.002, 0)$.

H Further extensions of the general SNCSTM

In this Web Appendix we sketch three extensions of the general SNCSTM described in Web Appendix A. First, we allow the causal effect of A_k to vary over time during an interval between exposure measurement times S_l and S_{l+1} (for $l \ge k$). Second, we allow the causal effect of a continuous exposure A_k to be non-linear in A_k. In particular, we shall sketch the method for a quadratic effect. Third, we have focused thus far on continuous and binary exposures. We shall now consider a categorical exposure with unordered categories.

H.1 Allowing the causal effect to vary with time during an interval between exposure measurement times

The SNCSTM of equation (2) assumes that the additive effect, $\psi_{k(l)}^\top Z_{k(l)}$ of A_k on the hazard between times S_l and S_{l+1} is constant over that time interval. If we instead allow $Z_{k(l)} = Z_{k(l)}(t)$ to be a function of t, then a modified version of equation (2) holds. Let us suppose, for example, that we assume $Z_{k(l)}(t) =$

$(L_k^\top, L_k^\top t)^\top$ and write $\psi_{k(l)}$ as $\psi_{k(l)} = (\psi_{k(l)}^{(L)\top}, \psi_{k(l)}^{(L2)\top})^\top$. Then

$$\begin{aligned}
&\frac{P\{T(\bar{A}_{k-1}, 0) \geq t \mid \bar{A}_k, \bar{L}_k, \bar{S}, T \geq S_k\}}{P\{T(\bar{A}_k, 0) \geq t \mid \bar{A}_k, \bar{L}_k, \bar{S}, T \geq S_k\}} \\
&\quad = \exp\left[\sum_{j=k}^{l-1} \left\{A_k L_k^\top \psi_{k(j)}^{(L)} (S_{j+1} - S_j) + A_k L_k^\top \psi_{k(j)}^{(L2)} (S_{j+1} - S_j)^2\right\}\right. \\
&\qquad \left. + A_k L_k^\top \psi_{k(l)}^{(L)} (t - S_l) + A_k L_k^\top \psi_{k(l)}^{(L2)} (t - S_l)^2\right]
\end{aligned}$$

when $S_l \leq t < S_{l+1}$. Note that the $(S_{j+1} - S_j)^2$ and $(t - S_l)^2$ terms arises because $\int_0^t u\, du = t^2$, just as the $(S_{j+1} - S_j)$ and $(t - S_l)$ terms arises because $\int_0^t 1\, du = t$. Now, when fitting the GLM for A_k given $\bar{A}_{k-1}$, $\bar{L}_k$ and $T \geq t$ (for $S_l \leq t < S_{l+1}$), we need to include interactions between L_k and $(t - S_l)$ and between L_k and $(t - S_l)^2$. Aside from the inclusion of these two interaction terms, the fitting of the GLM for A_k is the same as described in Web Appendix B. If the measurements are irregular (i.e. $\bar{S}$ varies from one individual to another), we also need to include other covariates, as described in Web Appendix B.2.

The way in which the gamma GLM is subsequently fitted to estimate $\psi_{k(l)}$ remains the same as was described in Web Appendix B.

H.2 Allowing the causal effect of A_k to be a non-linear function of A_k

We shall concentrate on the situation where exposure has a quadratic effect and the measurements are regular (and with $S_1, S_2, \ldots, S_{K+1}$ being multiples of δ). We have assumed thus far that Z_k is not a function of A_k. Now we let $Z_{k(l)} = (Z_{k(l)}^{*\top}, A_k)^\top$, where $Z_{k(l)}^*$ is a function of $\bar{A}_{k-1}$ and $\bar{L}_k$. We shall write $\psi_{k(l)}$ as $\psi_{k(l)} = (\psi_{k(l)1}^\top, \psi_{k(l)2})^\top$, with $\psi_{k(l)1}$ and $\psi_{k(l)2}$ corresponding to $Z_{k(l)}^*$ and A_k, respectively. We now have $h_{T(A_{k-1},0)}(t \mid \bar{A}_k, \bar{L}_k) = h_{T(A_k,0)}(t \mid \bar{A}_k, \bar{L}_k) - A_k Z_{k(l)}^{*\top} \psi_{k(l)1} - A_k^2 \psi_{k(l)2}$ for $S_l \leq t < S_{l+1}$.

Assume that

$$A_k \mid \bar{A}_{k-1}, \bar{L}_k, T \geq S_k \sim N(\alpha_{k0}^\top H_k, \tau_k^{-1}),$$

where $H_k = H_k(\bar{A}_{k-1}, \bar{L}_k)$ is a function of $\bar{A}_{k-1}$ and $\bar{L}_k$.

Then, for $S_k < t \leq S_{k+1}$, it follows from Model (2) and Bayes' Theorem that

$$A_k \mid \bar{A}_{k-1}, \bar{L}_k, T(\bar{A}_k, 0) \geq t \sim N\left(\frac{\alpha_{k0}^\top H_k - \psi_{k(k)1}^\top Z_{k(l)}^*(t - S_k)}{\tau_k + 2\psi_{k(k)2}(t - S_k)}, \frac{1}{\tau_k + 2\psi_{k(k)2}(t - S_k)}\right).$$

We can write this in the form

$$A_k \mid \bar{A}_{k-1}, \bar{L}_k, T(\bar{A}_k, 0) \geq t \sim N(\alpha_{k(k)1}(t)^\top H_k + \alpha_{k(k)2}(t)^\top Z_{k(l)}^*,\ \tau_{k(k)}(t)^{-1}).$$

where

$$\alpha_{k(k)1}(t) = \frac{\alpha_{k0}}{\tau_k + 2\psi_{k(k)2}(t - S_k)}$$

and

$$\alpha_{k(k)2}(t) = -\frac{\psi_{k(k)1}(t - S_k)}{\tau_k + 2\psi_{k(k)2}(t - S_k)}.$$

In particular, we have

$$A_k \mid \bar{A}_{k-1}, \bar{L}_k, T(\bar{A}_k, 0) \geq S_{k+1} \sim N(\alpha_{k(k)1}^\top H_k + \alpha_{k(k)2}^\top Z_{k(l)}^*,\ \tau_{k(k)}^{-1}),$$

where $\alpha_{k(k)1} = \alpha_{k(k)1}(S_{k+1})$ and $\alpha_{k(k)2} = \alpha_{k(k)2}(S_{k+1})$ and $\tau_{k(k)} = \tau_{k(k)}(S_{k+1})$. Similarly, for $S_{k+1} < t \leq S_{k+2}$,

$$A_k \mid \bar{A}_{k-1}, \bar{L}_k, T(\bar{A}_k, 0) \geq t \sim N\left(\frac{\alpha_{k(k)1}^\top H_k + \alpha_{k(k)2}^\top Z_{k(l)}^* - \psi_{k(k+1)1}^\top Z_{k(l)}^*(t - S_{k+1})}{\tau_{k(k)} + 2\psi_{k(k+1)2}(t - S_{k+1})}, \frac{1}{\tau_{k(k)} + 2\psi_{k(k+1)2}(t - S_{k+1})}\right)$$

which we can write in the form

$$A_k \mid \bar{A}_{k-1}, \bar{L}_k, T(\bar{A}_k, 0) \geq t \sim N\big(\alpha_{k(k+1)1}(t)^\top H_k + \alpha_{k(k+1)2}(t)^\top Z_{k(l)}^*,\ \tau_{k(k+1)}(t)^{-1}\big).$$

Generalising this, we see that we can estimate $E\{A_k \mid \bar{A}_{k-1}, \bar{L}_k, T(\bar{A}_k, 0) \geq t\}$ for any fixed $t \in [S_l, S_{l+1}]$ by linearly regressing A_k on H_k and $Z_{k(l)}^*$. Also, we can estimate $E\{A_k^2 \mid \bar{A}_{k-1}, \bar{L}_k, T(\bar{A}_k, 0) \geq t\}$ for any fixed $t \in [S_l, S_{l+1}]$ by using the relation

$$E\{A_k^2 \mid \bar{A}_{k-1}, \bar{L}_k, T(\bar{A}_k, 0) \geq t\} = \big[E\{A_k \mid \bar{A}_{k-1}, \bar{L}_k, T(\bar{A}_k, 0) \geq t\}\big]^2 + \tau_{k(l)}(t)^{-1}$$

and substituting in the estimate of $\tau_{k(l)}(t)^{-1}$ from the same linear regression. We shall denote the resulting estimates of $E\{A_k \mid \bar{A}_{k-1}, \bar{L}_k, T(\bar{A}_k, 0) \geq t\}$ and $E\{A_k^2 \mid \bar{A}_{k-1}, \bar{L}_k, T(\bar{A}_k, 0) \geq t\}$ as $\hat{E}\{A_k \mid \bar{A}_{k-1}, \bar{L}_k, T(\bar{A}_k, 0) \geq t\}$ and $\hat{E}\{A_k^2 \mid \bar{A}_{k-1}, \bar{L}_k, T(\bar{A}_k, 0) \geq t\}$. We propose that the linear regression model be fitted entirely separately for each value of $t = S_l, S_l + \delta, S_l + 2\delta, \ldots, S_{l+1}$. Just as in Web Appendix B, the weights $w_k(t)$ defined by equation (3) are needed when fitting the linear regressions with $l > k$.

The gamma GLM is fitted just as in Web Appendix B, but with covariates $-Z_{k(l)}^*[A_k - \hat{E}\{A_k \mid \bar{A}_{k-1}, \bar{L}_k, T(\bar{A}_k, 0) \geq Q\}]\delta$ and $-Z_{k(l)}^*[A_k^2 - \hat{E}\{A_k^2 \mid \bar{A}_{k-1}, \bar{L}_k, T(\bar{A}_k, 0) \geq Q\}]\delta$ in place of $-Z_{k(l)}\hat{\Delta}_k(Q)\delta$.

H.3 Categorical exposure with more than 2 levels

Suppose A_k is a categorical exposure with $J+1$ levels and we use a polychotomous regression model for A_k given $\bar{A}_{k-1}$, $\bar{L}_k$, $\bar{S}$ and $t \geq S_k$, i.e.

$$P(A_k = a \mid \bar{A}_{k-1}, \bar{L}_k, \bar{S}, T \geq S_k) = \frac{\exp(\alpha_{ka}^\top H_k)}{\sum_{j=0}^{J} \exp(\alpha_{kj}^\top H_j)}, \tag{21}$$

for $a = 0, \ldots, J$. Here $H_k = H_k(\bar{A}_{k-1}, \bar{L}_k, \bar{S})$ is a function of $\bar{A}_{k-1}$, $\bar{L}_k$ and $\bar{S}$, and the constraint $\alpha_{k0} = 0$ is imposed to ensure identifiability of parameters.

Suppose the SNCSTM is written as

$$\frac{P\{T(\bar{A}_{k-1}, 0) \geq t \mid \bar{A}_{k-1}, A_k = a, \bar{L}_k, \bar{S}, T \geq S_k\}}{P\{T(\bar{A}_k, 0) \geq t \mid \bar{A}_{k-1}, A_k = a, \bar{L}_k, \bar{S}, T \geq S_k\}} = \exp\left\{\sum_{j=k}^{l-1} A_k Z_{k(j)}^\top \psi_{k(j)}^{(a)} (S_{j+1} - S_j) + A_k Z_{k(l)}^\top \psi_{k(l)}^{(a)} (t - S_l)\right\} \tag{22}$$

for $a = 0, \ldots, J$ and with $\psi_k^{(0)} = 0$.

Define $\psi_k^{(a)}$ as $\psi_k^{(a)} = \left(\psi_{k(k)}^{(a)\top}, \psi_{k(k+1)}^{(a)\top}, \ldots, \psi_{k(K)}^{(a)\top}\right)$ and define $v_k(t, Z_k, \bar{S})$ as equal to $\left((t - S_k)Z_{k(k)}^\top, 0, \ldots, 0\right)^\top$ if $t \in [S_k, S_{k+1})$, as equal to $\left((S_{k+1} - S_k)Z_{k(k)}^\top, (t - S_{k+1})Z_{k(k+1)}^\top, 0, \ldots, 0\right)^\top$ if $t \in [S_{k+1}, S_{k+2})$, and as equal to $\left((S_{k+1} - S_k)Z_{k(k)}^\top, (S_{k+2} - S_{k+1})Z_{k(k+1)}^\top, (t - S_{k+2})Z_{k(k+2)}^\top, 0, \ldots, 0\right)^\top$ if $t \in [S_{k+2}, S_{k+3})$, etc.

Now, from equation (22) and Bayes' Rule, we have that for any $t > S_k$,

$$\begin{aligned} &P\{A_k = a \mid \bar{A}_{k-1}, \bar{L}_k, \bar{S}, T(\bar{A}_k, 0) \geq t\} \\ &\quad = \frac{\exp(\alpha_{ka}^\top H_k)\exp\{-v_k(t, Z_k, \bar{S})^\top \psi_k^{(a)}\}}{\sum_{j=0}^{J} \exp(\alpha_{kj}^\top H_k)\exp\{-v_k(t, Z_k, \bar{S})^\top \psi_k^{(j)}\}} \\ &\quad = \frac{\exp(\alpha_{ka}^\top H_k - v_k(t, Z_k, \bar{S})^\top \psi_k^{(a)}\}}{\sum_{j=0}^{J} \exp(\alpha_{kj}^\top H_k - v_k(t, Z_k, \bar{S})^\top \psi_k^{(j)}\}} \end{aligned} \tag{23}$$

Note that the constraints $\alpha_{k0} = \psi_k^{(0)} = 0$ mean that $\exp\{\alpha_{k0}^\top H_k - v_k(t, Z_k, \bar{S})^\top \psi_k^{(0)}\} = 0$.

Equation (23) implies that we can estimate $P\{A_k = a \mid \bar{A}_{k-1}, \bar{L}_k, \bar{S}, T(\bar{A}_k, 0) \geq t\}$ by fitting a polychotomous regression model for $P(A_k = a \mid \bar{A}_{k-1}, \bar{L}_k, \bar{S}, T \geq t)$ to the pseudo-individuals described in Web Appendix B with the covariates being H_k and $v_k(t, Z_k, \bar{S})$ and using weights $w_k(t)$ defined by equation (3) when $l > k$.

The gamma GLM is then fitted as described in Web Appendix B, but with $\hat{\Delta}_k(t)$ now defined as

$$\begin{bmatrix} I(A_k = 1) - P\{A_k = 1 \mid \bar{A}_{k-1}, \bar{L}_k, \bar{S}, T(\bar{A}_k, 0) \geq t\} \\ I(A_k = 2) - P\{A_k = 2 \mid \bar{A}_{k-1}, \bar{L}_k, \bar{S}, T(\bar{A}_k, 0) \geq t\} \\ \vdots \\ I(A_J = 1) - P\{A_k = J \mid \bar{A}_{k-1}, \bar{L}_k, \bar{S}, T(\bar{A}_k, 0) \geq t\} \end{bmatrix}$$

with $P\{A_k = a \mid \bar{A}_{k-1}, \bar{L}_k, \bar{S}, T(\bar{A}_k, 0) \geq t\}$ replaced by its estimate from the polychotomous regression model.

I Unbiasedness of gamma GLM estimating equation for fitting the point exposure multiplicative structural model at a single time point t

In Section 2 of our article, where we considered a point exposure, we described the method proposed by Dukes et al. (2018) for fitting the multiplicative structural model of equation (1) at a single time point t. Here we give a formal proof of the unbiasedness of the estimating equation corresponding to the gamma GLM described there, i.e. equation (3) in our article.

As $n \to \infty$, the estimated propensity score, $\hat{e}(L)$, converges to the true propensity score, $e(L) = E(A \mid L)$. Therefore, it suffices to show that the estimating function using the true propensity score, i.e.

$$\{A - e(L)\} \times \big(I(T \geq t) \exp[\psi\{A - e(L)\}t] - 1\big)$$

has expectation zero at the true value of ψ. We now show this.

First, we can write

$$\begin{aligned}
&E\big\{\{A - e(L)\} \times \big(I(T \geq t) \exp[\psi\{A - e(L)\}t] - 1\big)\big\} \\
&= E_L\big(\, E_A\big[\, E_T\big\{\{A - e(L)\} \times \big(I(T \geq t) \exp[\psi\{A - e(L)\}t] - 1\big) \mid A, L\big\} \mid L\,\big]\,\big) \\
&= E_L\big[\, E_A\big\{\, \{A - e(L)\}\, E_T\big(I(T \geq t) \exp[\psi\{A - e(L)\}t] - 1 \mid A, L\big) \mid L\,\big\}\big]
\end{aligned} \tag{24}$$

Next, we can write

$$\begin{aligned}
&E_T\big(I(T \geq t) \exp[\psi\{A - e(L)\}t] - 1\big) \mid A, L\big) \\
&= \exp\{-\psi e(L)t\}\ \exp(\psi A t)\ E_T\{I(T \geq t) \mid A, L\} - 1 \\
&= \exp\{-\psi e(L)t\}\ \exp(\psi A t)\ P(T \geq t \mid A, L) - 1 \\
&= \exp\{-\psi e(L)t\}\ P\{T(0) \geq t \mid A, L\} - 1 && (25) \\
&= \exp\{-\psi e(L)t\}\ P\{T(0) \geq t \mid L\} - 1 && (26)
\end{aligned}$$

Lines (25) and (26), follow from, respectively, the multiplicative structural model and the no unmeasured confounders assumption that $T(0) \perp\!\!\!\perp A \mid L$.

From equations (24) and (26), we have

$$\begin{aligned}
&E\big\{\, \{A - e(L)\} \times \big(I(T \geq t) \exp[\psi\{A - e(L)\}t] - 1\big)\,\big\} \\
&= E_L\big(\, E_A\big[\, \{A - e(L)\} \times \exp\{-\psi e(L)t\}\ P\{T(0) \geq t \mid L\} - 1 \mid L\,\big]\,\big) \\
&= E_L\big(\, [\exp\{-\psi e(L)t\}\ P\{T(0) \geq t \mid L\} - 1] \times E_A\big\{A - e(L) \mid L\big\}\,\big) \\
&= E_L\big(\, [\exp\{-\psi e(L)t\}\ P\{T(0) \geq t \mid L\} - 1] \times\ \{e(L) - e(L)\}\,\big) \\
&= 0,
\end{aligned}$$

as required.

J Estimating equations for SNCSTM with effect modification

At the beginning of Section 5 of our article we described an example of a SNCSTM where the causal effects of A_0 and A_1 are modified by, respectively, L_0 and L_1, and described how to fit this model. Here we give the estimating equations for each of the three gamma GLMs used to estimate, respectively, (ψ_1^0, ψ_1^L), $(\psi_{0(0)}^0, \psi_{0(0)}^L)$ and $(\psi_{0(1)}^0, \psi_{0(1)}^L)$ in this model.

The contribution of a pseudo-individual to the estimating equations of the gamma GLM for estimating (ψ_1^0, ψ_1^L) is

$$\{A_1 - \hat{e}_1(A_0, \bar{L}_1, Q)\} \times \big(I(T \geq Q + \delta)\ \exp[\{A_1 - \hat{e}_1(A_0, \bar{L}_1, Q)\}(\psi_1^0 + L_1\psi_1^L)\delta] - 1\big).$$

Similarly, the contribution of a pseudo-individual to the estimating equations of the gamma GLM for estimating $(\psi_{0(0)}^0, \psi_{0(0)}^L)$ is

$$\{A_0 - \hat{e}_0(L_0, Q)\} \times \big(I(T \geq Q + \delta)\ \exp[\{A_0 - \hat{e}_0(L_0, Q)\}(\psi_{0(0)}^0 + L_0\psi_{0(0)}^L)\delta] - 1\big).$$

As described in Section 5 of our article, the gamma GLM for estimating $(\psi_{0(1)}^0, \psi_{0(1)}^L)$ uses weights $\exp\{A_1(\hat{\psi}_1^0 + L_1\hat{\psi}_1^L)(Q - s_1)\}$ and the modified outcome variable $I(T \geq Q + \delta)\exp\{A_1(\hat{\psi}_1^0 + L_1\hat{\psi}_1^L)\delta\}$. The contribution of a pseudo-individual to the estimating equations of this gamma GLM is

$$\begin{aligned}&\exp\{A_1(\hat{\psi}_1^0 + L_1\hat{\psi}_1^L)(Q - s_1)\} \times \{A_0 - \hat{e}_0(L_0, Q)\}\\ &\quad\times\big(I(T \geq Q + \delta)\ \exp\left[A_1(\hat{\psi}_1^0 + L_1\hat{\psi}_1^L)\delta + \{A_0 - \hat{e}_0(L_0, Q)\}(\psi_{0(1)}^0 + L_0\psi_{0(1)}^L)\delta\right] - 1\big).\end{aligned}$$

References

[1] SR Seaman, O Dukes, RH Keogh, and S Vansteelandt. Adjusting for time-varying confounders in survival analysis using structural nested cumulative survival time models. *Biometrics*, 76(2):472–483, 2020.
